# Targeting platelet-tumor cell interactions via thromboxane A_2_-prostanoid receptor blockade to reduce metastasis in triple negative breast cancer

**DOI:** 10.1186/s40164-025-00723-7

**Published:** 2025-11-13

**Authors:** Veeresh Toragall, Ann C. Rester, Salma Begum, Oluwaseyi T. Shofolawe-Bakare, Kenneth Hulugalla, Jerry D. Monroe, John P. Bentley, Yann Gibert, Thomas A. Werfel

**Affiliations:** 1 Department of Biomedical Engineering, University of Mississippi, University, MS 38677 USA; 2 Department of Chemical Engineering, University of Mississippi, University, MS 38677 USA; 3 Department of BioMolecular Sciences, University of Mississippi, University, MS 38677 USA; 4 Department of Cell and Molecular Biology, Cancer Center and Research Institute, University of Mississippi Medical Center, Jackson, MS 39216 USA; 5 Cancer Center and Research Institute, University of Mississippi Medical Center, Jackson, MS 39216 USA; 6 Department of Pharmacy Administration, University of Mississippi, University, MS 38677 USA; 7 Present Address: Department of Medical and Molecular Genetics, Indiana University, 675 W. Walnut Street, Indianapolis, IN 46202 USA

**Keywords:** Ifetroban, Platelet activation, Thromboxane receptor and antimetastatic efficacy

## Abstract

In addition to their central role in blood hemostasis, it is increasingly clear that platelets contribute to multiple steps in the metastatic cascade. Platelets are one of the most abundant cells with which tumor cells interact once they enter the circulation, and the interaction of platelets with tumor cells can improve tumor cell survival, arrest and adhesion at secondary sites, and extravasation. Therefore, targeting the interaction between platelets and circulating tumor cells could be an effective approach for reducing metastasis. Here, we repurpose the thromboxane A_2_-prostanoid receptor (TPr) inhibitor, ifetroban, to block platelet-tumor cell interactions and reduce metastasis in models of triple negative breast cancer (TNBC). We utilize in vitro co-culture models of platelets and tumor cell lines to assess the impact of ifetroban treatment on the adhesion of platelets to tumor cells. In each case, platelet-tumor cell adhesion was significantly increased when the TPr agonist U46619 was introduced, while pre-treatment with ifetroban (TPr antagonist), significantly reduced platelet-tumor cell adhesion. Further, we used a zebrafish model system to rapidly assess metastasis and platelet interactions in vivo, showing that ifetroban reduces metastasis of MDA-MB-231 xenografts without reducing platelet number in CD41 transgenic zebrafish embryos. Finally, we confirm that ifetroban significantly reduces both lung and liver metastasis in multiple murine models of TNBC (4T1 and MDA-MB-231). In these models, we observed that ifetroban reduces metastasis in the absence of a primary tumor and when TPr is deleted from tumor cells, further supporting the notion that ifetroban attenuates the supportive role of platelet TPr in the metastatic cascade. Based on the results of this study, ifetroban could be pursued as a clinical agent to reduce metastasis in TNBC patients.

**Graphical Abstract:**

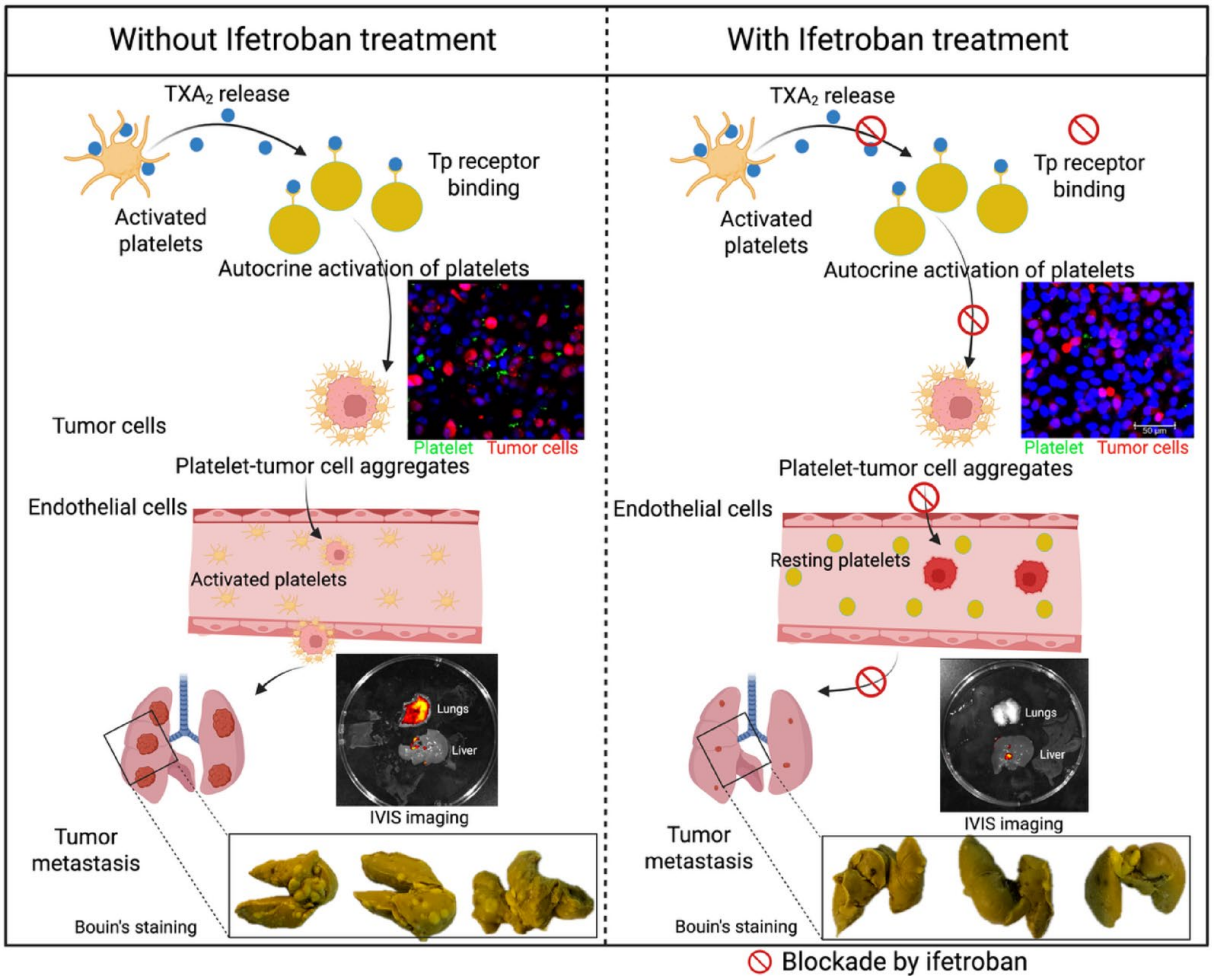

**Supplementary Information:**

The online version contains supplementary material available at 10.1186/s40164-025-00723-7.

## Introduction

Triple negative breast cancer (TNBC) remains a difficult-to-treat malignancy because of its aggressiveness and the lack of sufficient treatment options that exist [1]. While cytotoxic agents are available and are effective in many cases, there is a dearth of pharmaceutical agents that directly interfere with the pathological mechanisms of TNBC [2]. For instance, although many TNBCs are highly metastatic, there are no agents in use that directly interfere with metastatic dissemination of the cancer [3]. Agents that directly block the spreading of tumor cells by inhibiting specific events in the metastatic cascade could reduce the mortality associated with metastasis [4]. Moreover, these agents could complement or even synergize with existing cytotoxic and immune therapies. One route of metastasis which could be targeted to prevent cancer dissemination, but which remains understudied, is platelet-mediated metastasis, whereby platelets help tumor cells navigate the metastatic cascade by supporting their survival and spread to secondary tissues [5].

As metastasizing tumor cells intravasate into the vasculature, platelets are one of the most pervasive cell types with which they interact [6, 7]. Platelets can produce signals that promote tumor cell epithelial-mesenchymal-like transition (EMT) and migration across endothelium at the primary tumor and metastatic sites [8, 9]. Platelets form a steric barrier on the surface of tumor cells that prevents their recognition and clearance by immune cells (e.g. NK cells, T cells, neutrophils, and macrophages) in the circulation [8, 10]. Platelets may also reduce circulating tumor cell (CTC) anoikis through integrin-mediated survival signaling [11, 12]. To support extravasation at distant sites (e.g. lungs), platelets increase the affinity of selectin-based interactions that support tumor cell arrest [13, 14] and integrin-based interactions that support adhesion along the endothelium [15, 16]. As a key source of circulating angiogenesis-related factors, platelets regulate processes of tumor angiogenesis and vascular integrity [17–19]. The process of tumor cell-mediated platelet aggregation/activation can be either direct or indirect activation once the tumor cells enter circulation. Direct activation occurs through receptor interactions on the platelet and CTC membranes, such as P-selectin, integrins, glycoproteins with PSGL-1 (P-selectin glycoprotein ligand-1), and metalloproteinase domain-containing protein 9 (ADAM9) [10, 13]. Indirect activation is triggered by soluble metabolites such as adenosine diphosphate (ADP), thrombin, and thromboxane A2 (TXA_2_), which stimulate platelet activation [20, 21].

One such source of indirect activation, TPr, is known to stimulate platelet aggregation when activated by its ligand, TXA_2_ [22] and/or synthetic agonists such as U46619 [23]. The TPr is widely expressed in various tissues and cell types, with expression levels varying significantly. High expression occurs in platelets, vascular smooth muscle, and lung tissue. Moderate expression is found in endothelial cells, renal tissue, and macrophages. Low-to-moderate expression appears in cardiomyocytes and immune organs like the spleen and thymus. TPr exists in two isoforms TPrα and TPrβ which are differentially expressed across these cells and tissues. This underscores the important role of TPr in health and disease [24–26]. More recently, an appreciation that TPr could contribute to the spread of cancer via its multifaceted roles in the vasculature is developing. Cell and animal models of metastasis implicate TXA_2_ in metastatic disease through several key metastatic steps such as tumor cell motility and angiogenesis [27]. *TBXA2R*-null platelets aggregate poorly with tumor cells when co-cultured ex vivo, fail to accumulate at sites of lung metastasis, and express lower levels of P-selectin and integrins, resulting in reduced metastases after intravenous injection of B16-F1 melanoma cells in *TBXA2R*-null animals [28]. Recent studies also highlight a role for COX-1 in the generation of a pre-metastatic niche within the vasculature, in large part through its ability to increase production of TXA_2_, the TPr ligand, enhancing the formation of platelet-tumor cell aggregates upon the vascular wall to facilitate metastatic seeding [29]. Additionally, it has been shown that TXA_2_ mediates immunosuppression of T cell responses, a property that can be reversed by inhibiting the TXA_2_ signaling axis [26]. Finally, direct blockade of TXA_2_ synthase or TPr disrupts TXA_2_ signaling and reduces metastasis in experimental models of multiple cancer types [22, 30].

Here, we tested if ifetroban, a potent and highly specific antagonist of TPr originally developed for cardiovascular indications [31], could be redeployed to block the metastasis of TNBCs. Importantly, the safety of ifetroban in humans is well established based on multiple clinical trials and the over 1,400 patients total dosed with ifetroban to date [31, 32]. Safety pharmacology studies reveal no evidence of severe adverse effects in a wide variety of models targeting central nervous, cardiovascular, respiratory, digestive, renal, and motor control systems. To test the hypothesis that ifetroban reduces metastasis by inhibiting platelet-tumor cell interactions, we first studied platelet activation and platelet-tumor cell adhesion in cellular co-culture models. Next, we used zebrafish models to image TNBC cell dissemination and platelet interactions in a living system. Lastly, we monitored platelet activation, CTC counts, and tumor growth and metastasis in mice harboring orthotopic xenografts of the 4T1 and MDA-MB-231 TNBC cell lines.

## Materials and methods

### Cell culture and maintenance

RFP+-expressing human TNBC cells (MDA-MB-231-RFP, RRID: CVCL_JZ06) and murine breast cancer cells (4T1, RRID: CVCL_0125) were purchased from the American Type Culture Collection (ATCC) and the *TBXA2R* knockout (TPr KO) cell lines were purchased from EDITGENE (CA, USA). Cell cultures were routinely tested for mycoplasma infection and maintained in DMEM (Gibco, Cat# 11965092) supplemented with 10% heat-inactivated FBS (Gibco, Cat# 10099141) and antibiotic–antimycotic (1%) (Gibco, Cat# 15240062). Cells were maintained at 37 °C in a humidified atmosphere with 5% CO_2_. The culture medium was changed every 2–3 days. All experiments were conducted on cells between passages 5 to 13.

### Platelet and tumor cell adhesion

Tumor cell-platelet binding was visualized using confocal microscopy (LEICA SP8X, Wetzlar, Germany) as per our previously optimized method [33]. Briefly, CellTracker™ Green-labeled platelets (5*10^7^ in 100 µL cell culture media or platelet-poor plasma) were added to 85–90% confluent tumor cells (MDA-MB-231-RFP) grown in a 35 mm collagen-treated cover glass (MatTek Corporation, catalog number: NC9125998). Cells were treated with U46619 (0.3 µM), ifetroban (10 µM) re-constituted in phosphate-buffered saline (PBS, Gibco, Cat# 10010023), or both in combination at 37 °C for 30 min. After washing thrice with PBS, cells were fixed with 2% paraformaldehyde and mounted with DAPI-containing mounting media (Vector Laboratories). A series of treatment methods were designed to assess the individual and combined effects of ifetroban on platelet-tumor cell interactions, allowing us to determine the most effective approach for enhancing treatment outcomes. Hence, we have used three treatment methods: (1) pre-treating the platelets with ifetroban and vehicle, (2) co-treating the platelets and tumor cells with ifetroban and vehicle, or (3) pre-treating the tumor cells with ifetroban and vehicle. The area of each well was monitored by confocal microscopy (LEICA SP8, Wetzlar, Germany) [33, 34].

Tumor cells and platelets were pretreated with vehicle, U46619 (0.3 µM), ifetroban (10 µM), or both at 37 °C for 30 min, washed twice with PBS or washing buffer, and co-incubated. Tumor cells and platelets were collected after treatment and incubated with an anti-CD61-APC antibody (336412, BioLegend, RRID: AB_10707694, 104316, BioLegend, RRID: AB_2561734) for 30 min at 4 °C [29]. Samples were then fixed with 0.01% neutral buffered formalin for 30 min at room temperature or overnight at 4 °C. The samples were centrifuged at 400 g for 20 min at 18 °C to remove the unstained antibodies and washed thrice with PBS. Finally, the pellet was reconstituted with FACS buffer (1:10 dilution) and the suspension was analyzed with an Attune NxT flow cytometer (ThermoFisher). Platelet aggregation was indicated by the depletion of the single-platelet population as indicated by increased side scattering and CD61 expression [29, 35].

### Plate reader

To quantify the tumor cell-platelet interactions, MDA-MB-231-RFP cells are grown to 85–90% confluence in a 48-well plate. Platelets (5*10^7^ in 100 µL PPP) labeled with CellTracker™ Green were pretreated with U46619, ifetroban or both and then added to the tumor cell cultures. After 30 min incubation, unbound platelets were removed by washing two times with PBS. Platelets adhered to tumor cells were lysed with 100 µL of 10% Triton X-100 (diluted in PBS) and transferred to a black 96-well plate. Green fluorescence was quantified at an excitation wavelength of 485 nm and an emission wavelength of 520 nm using a microplate reader (Agilent) [33, 34].

###  In vivo zebrafish studies

Transparent *casper*(*roy*^−^/^−^;*nacre*^−^/^−^) and Tg(CD41:GFP) transgenic zebrafish lines were maintained at 28 °C with a 14 h light and 10 h dark cycle according to established protocols [36]. All zebrafish experiments were conducted in accordance with the guidelines of the animal care and use committee of the University of Mississippi Medical Center (UMMC) Jackson, MS, USA, and approved by the UMMC institutional biosafety and IACUC review committees (IACUC protocol number: 2021 − 1161). TNBC xenografts were performed as previously described [37]. Briefly, zebrafish larvae at 2 days post fertilization (dpf) were anesthetized with 0.2 mg/ml tricaine methanesulfonate (MS-222) (Sigma-Aldrich, St. Louis, MO), placed in a glass injection tray with E3 water (5 mM NaCl, 0.17 mM KCl, 0.33 mM CaCl_2_, 0.33 mM MgSO_4_, 1 ppm methylene blue)(Sigma) and approximately 100 cells treated with the red fluorescent dye, DiI [37], were then injected into the yolk sac of individual larvae using a Tritech Research (Los Angeles, CA) microinjector and Labomed Luxeo 6z (Fremont, CA) stereo microscope. Ifetroban stock solution was prepared in DMSO at a concentration of 10 mM. For zebrafish experiments, ifetroban was used at a final concentration of 5.25 µM while control was exposed to DMSO. The concentration of 5.25 µM for ifetroban exposure and 10^− 7^ M for U46619 treatment of zebrafish embryos were selected after dose-response analysis. Zebrafish images were taken with an Axioimager 2.0 (Zeiss) using an Axiocam 705 color digital camera (Zeiss). Photos were processed with Adobe Photoshop software (RRID: SCR_014199).

###  In vivo mouse studies

All procedures were performed in compliance with the Institutional Animal Care and Use Committee (IACUC, protocol number: 23 − 007)–approved protocol of the University of Mississippi and following National Institutes of Health (NIH) guidelines. Animals were quarantined in the University of Mississippi’s animal house for 7 days with a 12-hour dark/light cycle. During this period, they had free access to standard pellet feed and water and were monitored daily by veterinary staff. Balb/C (female, 5–6 weeks old, 14–17 g) and athymic nude mice (female, 5–6 weeks old, 16–19 g) were purchased from Inotiv, Inc. Mice were randomly segregated into two experimental groups: Ifetroban and Control. Ifetroban was suspended in 4% sucrose and administered once daily at 50 mg/kg via oral gavage (starting two weeks before tumor implantation or injection and continuing until the end of study). The dose of ifetroban used in murine studies was translated from the human dose being used in antimetastatic clinical trials; the human dose of 250 mg (~ 4.03 mg/kg) was multiplied by 12.3 for the murine dose of 50 mg/kg [38]. For orthotopic tumor implantation, 1*10^6^ 4T1 or MDA-MB-231-RFP cells were suspended in a 1:1 mixture of Matrigel: PBS and injected into the 4th inguinal mammary fat pad of Balb/C (4T1/TPr KO 4T1) or athymic nude (MDA-MB-231-RFP) mice [27]. For the experimental lung metastasis model, 5 × 10^5^ 4T1 cells suspended in 100 µl PBS were intravenously injected into the tail vein of Balb/C mice. During the treatment period, the change in animal weight and the tumor volume were noted every three days. Tumor dimensions were measured using digital calipers, and tumor volume was calculated as: volume = length × width^2 × 0.52. At the end of the study, blood, tumors, and vital organs (liver and lungs) from animals were collected and analyzed. Animals were terminated when tumors reached 1.2 cm^3^ in size or body weight loss exceeded 20% of the original body weight.

### Isolation and quantification of CTCs

At the experimental endpoint, blood was collected from mice in each treatment group using blood collection tubes coated with EDTA (Greiner Bio-One, USA). As per the manufacturer’s instructions, whole blood was diluted with PBS (1:1 *v/v*), added to the Ficoll-Paque PLUS media solution (GE Healthcare), and centrifuged at 420 g for 32 min at 18 °C. The mononuclear cell layers (PBMCs) were isolated and the fluorescence intensity of RFP+ cells was quantified at an excitation wavelength of 555 nm and an emission wavelength of 585 nm using a Synergy H1 microplate reader (Agilent) [29, 33, 34].

###  In vivo imaging of tumor growth and metastasis

Organs including the lungs, liver, and tumors were harvested from athymic nude mice harboring MDA-MB-231-RFP orthotopic xenografts at the study endpoint. These organs were imaged ex vivo at an excitation/emission wavelength of 555/585 nm and an exposure time of 5 s using a Lumina III IVIS (Revvity, Inc.).

###  Whole blood analysis

At the end of the experiment, blood samples were collected in K2EDTA tubes (Greiner Bio-One, USA) via cardiac puncture for whole blood analysis using Sysmex XTV automated hematology analyzers (Sysmex, Mundelein IL, USA). The blood levels of white blood cells, neutrophils, red blood cells, hemoglobin, lymphocytes, monocytes, eosinophils, mean corpuscular hemoglobin, and platelets were measured and analyzed.

### Hematoxylin and Eosin (H&E) staining

Paraffin sections were dewaxed using xylene and then hydrated with ethanol solutions of decreasing concentrations (100%-50%). Following hydration, the sections were subjected to hematoxylin staining and washing with 1% HCl, followed by 70% ethanol and immersing in 1% NH_4_OH for blue color development. This was followed by Eosin staining for 30 s, and the stained sections were then dehydrated again through a series of ethanol solutions (95% and 100%) to clear the xylene before being mounted. The H&E-stained sections were visualized, and images were captured using a Lionheart LX automated microscope (Agilent).

### Immunohistochemistry (IHC)

The tissue sections were deparaffinized using xylene, followed by rehydration using a series of graded alcohol solutions, and then transferred and rinsed twice with PBS. Antigen retrieval was performed with 0.01 M citrate buffer (pH 6.0) at 95 ℃ for 10 min. After cooling, the sections were treated with 3% hydrogen peroxide at room temperature for 10 min. Then, the sections were permeabilized in ultrapure water containing 0.2% Triton for 10 min and blocked with 10% goat serum in PBS for 1 h. The sections were then incubated with the primary antibodies, CD31 (1:100, # 776995, Cell Signaling, RRID: AB_2722705), Ki-67 (1:200, # PA5-19462, Invitrogen, RRID: AB_10981523) and CC3 (1:100, # PP229AA, Biocare) for at 4 ℃ overnight. Following three washes with PBS for 15 min each, the sections were incubated with GTVision™ III Detection System Mouse & Rabbit Kit (DAB) (GK500705, Gene Tech). Finally, the sections were stained with hematoxylin for 10 s to visualize nuclei. All images were observed and photographed via Lionheart LX automated light microscope (Agilent).

###  Statistical analysis

All data were analyzed using GraphPad Prism Version 10.1.1 (San Diego, USA, RRID: SCR_002798). Comparisons between the means of control and experimental groups were performed using a two-tailed, unpaired t-test. Values in Fig. 4 are presented as mean ± SEM due to the high sample size. All other values are presented as mean ± SD. One-way ANOVA was performed to assess statistical significance among three or more treatment groups, with Tukey’s post hoc test to determine statistical differences between specific groups. Survival analysis was performed, and groups were compared using the log-rank (Mantel-Cox) test. The number of mice for in vivo experiments is included in the appropriate figure legends. For analyses where the number of observations was *n* = 3 per group, sensitivity analyses were conducted with nonparametric procedures (i.e., median test and Kruskal-Wallis one-way ANOVA, both followed up with stepwise stepdown multiple comparisons) to assess robustness of the findings [39]. Significance levels were documented using the following symbols: NS, *P < 0.05, **P < 0.01, ***P < 0.001, and ****P < 0.0001.

**Fig. 1:**
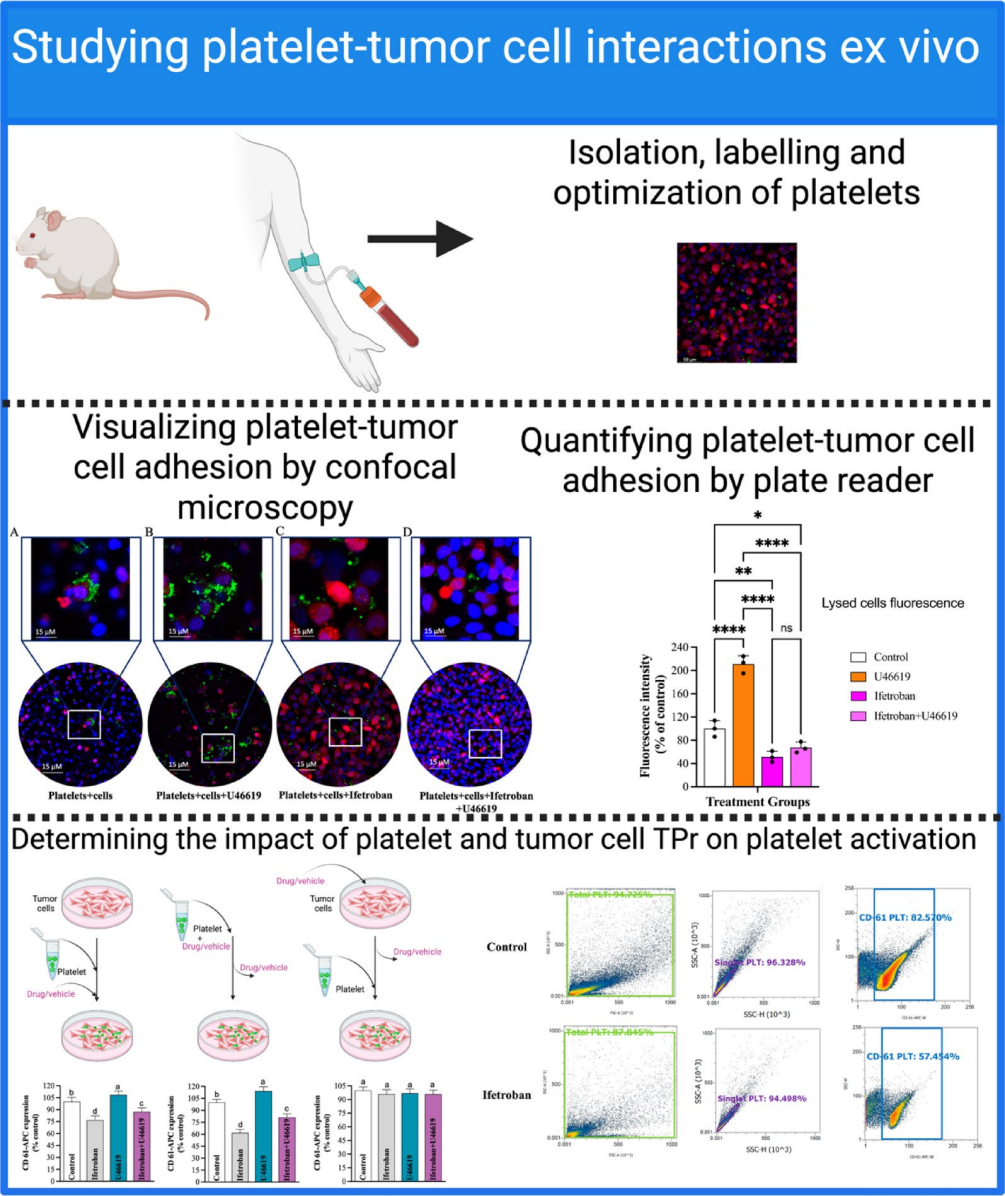
Overview of Ex-vivo platelet and tumor cell interaction studies

## Results

###  Ex-vivo platelet-tumor cell adhesion

**Fig. 2:**
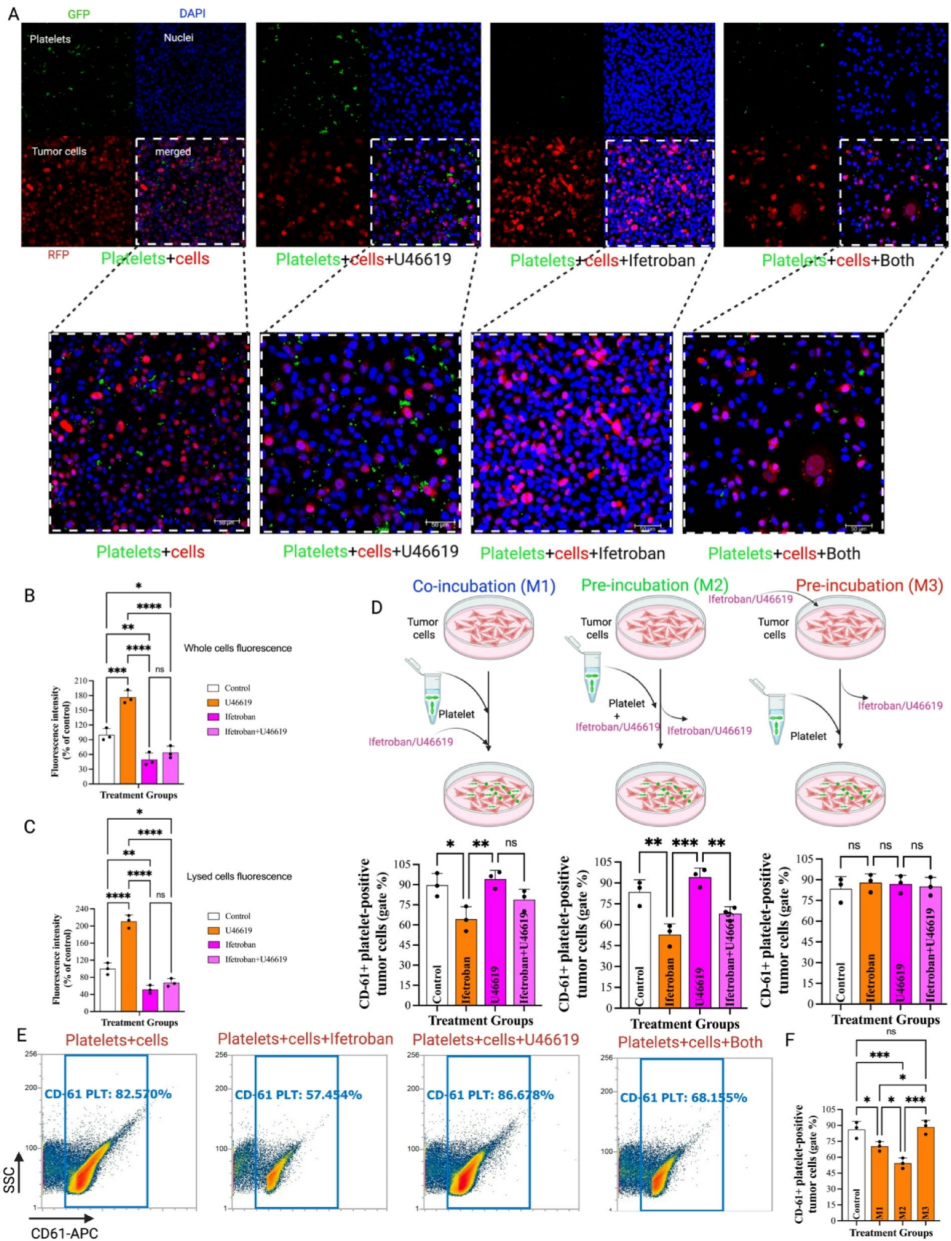
Ifetroban blocks platelet-tumor cell interactions ex vivo. **A** Representative confocal microscopy images showing the interactions between CMFDA-labeled platelets with RFP-tagged MDA-MB-231 tumor cell nuclei stained with DAPI under different treatment conditions. **B** Quantifying adherent platelets around tumor cells by measuring green fluorescence intensities of confocal images using Image J software. **C** Quantification of platelet (CMFDA–labeled) and tumor cell (MDA-MB-231-RFP) interactions using a microplate reader under different treatment conditions. **D** The percentage of tumor cells with CD61 + platelets bound to their surface was quantified by flow cytometry for three parallel treatment protocols: M1) Co-incubation of platelets, tumor cells, and ifetroban/U46619, M2) Pre-incubation of platelets and ifetroban/U46619 prior to co-culture with tumor cells, and M3) Pre-incubation of tumor cells and ifetroban/U46619 prior to co-culture with platelets. **E** Flow cytometry histograms of the M2 treatment protocol. **F** Comparison of CD-61 expression levels between M1, M2, and M3 treatment protocols. Data are represented as mean ± SD (*n* = 3) and analyzed by one-way ANOVA followed by post-Tukey’s test. The statistical significance is indicated by asterisks (*P < 0.05, **P < 0.01, ***P < 0.001 and ****P < 0.0001 vs. Control, NS-nonsignificant). Stepwise stepdown multiple comparisons following median tests and Kruskal-Wallis one-way ANOVA showed consistent findings with respect to the pattern of statistical significance among the groups

We first investigated the interactions of isolated human platelets and MDA-MB-231-RFP cells ex vivo to establish the role of TPr in platelet adhesion with breast cancer cells (Fig. 1). By visualizing CellTracker Green-labeled platelets and RFP+ MDA-MB-231-RFP cells using confocal microscopy, we noted that there was a substantial adhesion of platelets on the surface of MDA-MB-231-RFP cells in the absence of any external stimuli, indicating a propensity for intrinsic platelet-tumor cell interactions (Fig. 2A). Pre-treatment of platelets with the TPr agonist U46619 further increased platelet adhesion to MDA-MB-231-RFP cells by ~ 90% (Fig. 2A-B). Alternatively, pre-treatment with ifetroban reduced platelet-tumor cell adhesion by ~ 50% compared to untreated cells (Fig. 2A-B). Moreover, the co-incubation of U46619 and ifetroban led to a similar reduction in platelet-tumor cell adhesion, confirming that ifetroban can inhibit TPr signaling and prevent platelet-tumor cell adhesion even in the presence of activating signals. We further validated these results by washing and lysing the cells, followed by bulk quantification of green fluorescence from the labeled platelets on a microplate reader. These bulk fluorescence measurements indicated the same trends. U46619 treatment increased the number of platelets adhering to tumor cells in culture, which was abrogated and even reduced compared to untreated cells in the presence of the TPr inhibitor, ifetroban (Fig. 2C). The concentration of platelets was standardized and identical between the vehicle and ifetroban-treated samples in these studies. However, the possibility that treatment with ifetroban at 10 µM decreases platelet number cannot be entirely ruled out as a possible explanation of these experimental results.

We next sought to establish the cell-type dependence of TPr inhibition on platelet-tumor cell adhesion with a series of treatment protocols that would activate/inhibit TPr selectively in platelets, tumor cells, or both in combination by using CD-61 as a measure of platelet activation (Supplementary Fig. 1). Co-treatment of platelets and tumor cells with ifetroban, as well as pre-treatment of platelets with ifetroban, both resulted in decreased tumor cell-platelet interactions compared to untreated cells and in the presence of the TPr agonist U46619 (Fig. 2D-E). However, pre-treatment of tumor cells with ifetroban or U46619 prior to the addition of platelets had no effect on binding of platelets to tumor cells (Fig. 2D-E). Therefore, the blockade of platelet TPr by ifetroban is primarily responsible for reduced platelet-tumor cell interactions, and TPr blockade in tumor cells does not impact platelet-tumor cell adhesion. Comparing the levels of inhibition of tumor cell-platelet interactions between treatment protocols (co-incubation (M1), platelet pre-incubation (M2), and tumor cell pre-incubation (M3)), platelet pre-incubation with ifetroban had the largest inhibitory effect on binding of platelets to tumor cells (Fig. 2F). Whereas pre-incubation of tumor cells with ifetroban had no significant impact on binding of platelets to tumor cells. We also evaluated the impact of varying co-incubation durations (60, 90, and 120 min) between platelets and tumor cells on platelets adhesion status to ensure that platelets were not triggered by tumor cells at longer periods of co-incubation. We found that tumor cell pre-treatment with ifetroban followed by co-incubation of tumor cells and platelets for 60-, 90-, and 120 min still resulted in no significant tumor cell-platelet interaction in culture with the tumor cells (Supplementary Fig. 2).

### Ifetroban exposure does not reduce the number of platelets in zebrafish larvae

**Fig. 3:**
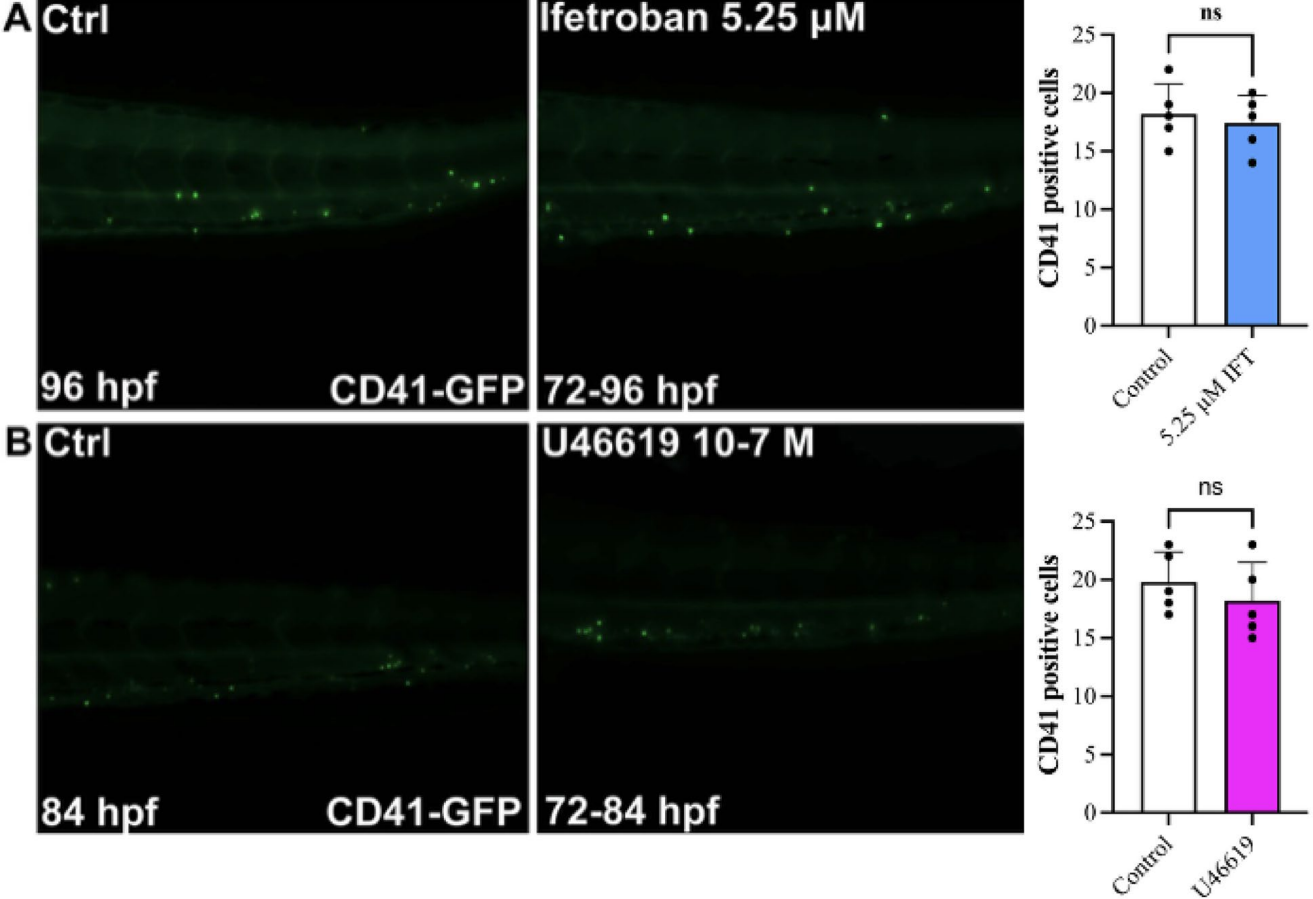
Ifetroban and U46619 treatments do not change CD41 levels in zebrafish embryos.** A** Representative images of CD41 transgenic zebrafish embryos treated with a control (left panel; image taken at 96 hpf) or 5.25 µM ifetroban treatment (right panel; treatment from 72–96 hpf with image taken at 96 hpf). **B** Representative images of CD41 transgenic zebrafish embryos treated with a control (left panel; image taken at 84 hpf) or 10^− 7^ M U46619 treatment (right panel; treatment from 72–84 hpf with image taken at 84 hpf). Data are represented as mean ± SD (*n* = 5). The statistical significance was analyzed using two-tailed, unpaired t-tests. hpf = hours post fertilization; ns = not significant; Ctrl = control

We next studied the effect of ifetroban exposure on the number of platelets in vivo to establish a safe working dose for zebrafish studies. Tg(CD41:GFP) transgenic zebrafish larvae expressing green fluorescent protein (GFP) under the control of the promoter of the CD41 gene is predominantly expressed in platelets in zebrafish larvae [40]. First, using zebrafish CD41:GFP transgenic larvae xenografted at 2 days post-fertilization (dpf) with TNBCs treated with red fluorescent DiI, we observed adhesion of platelets on the surface of xenografted MDA-MB-231 cells at 3 dpf (Supplementary Fig. 3), similarly to what we observed in vitro (Fig. 2A-B). We performed a dose-response study to identify the maximum tolerated dose at which platelet numbers are not impacted by ifetroban treatment. At doses of ifetroban up to 6.25 µM, we observed no reduction in CD41 + platelet numbers in Tg(CD41:GFP) transgenic zebrafish larvae (Supplementary Fig. 4). However, exceeding this dose began to impact platelet numbers. We observed a reduction of CD41 + platelets in zebrafish larvae treated with 7.5 µM ifetroban (Supplementary Fig. 5A). Moreover, CD41 + platelet numbers continued to deplete with time when exposed to 7.5 µM ifetroban from 78 to 96 h (Supplementary Fig. 5B-E). We then exposed Tg(CD41:GFP) embryos to 5.25 µM ifetroban from 72 h post-fertilization (hpf) onwards with microscopy being performed at 78, 84, or 96 hpf. We found that 6 and 12 h of ifetroban exposure did not affect platelet number (not shown) and that 24 h exposure to 5.25 µM ifetroban did not reduce CD41 positive cells (Fig. 3A, top line). Moreover, activation of TPr using the potent and selective agonist U46619 did not impact the number of platelets either (Fig. 3B, bottom line).

### Ifetroban exposure blocks the migration of xenografted TNBCs in zebrafish larvae

**Fig. 4:**
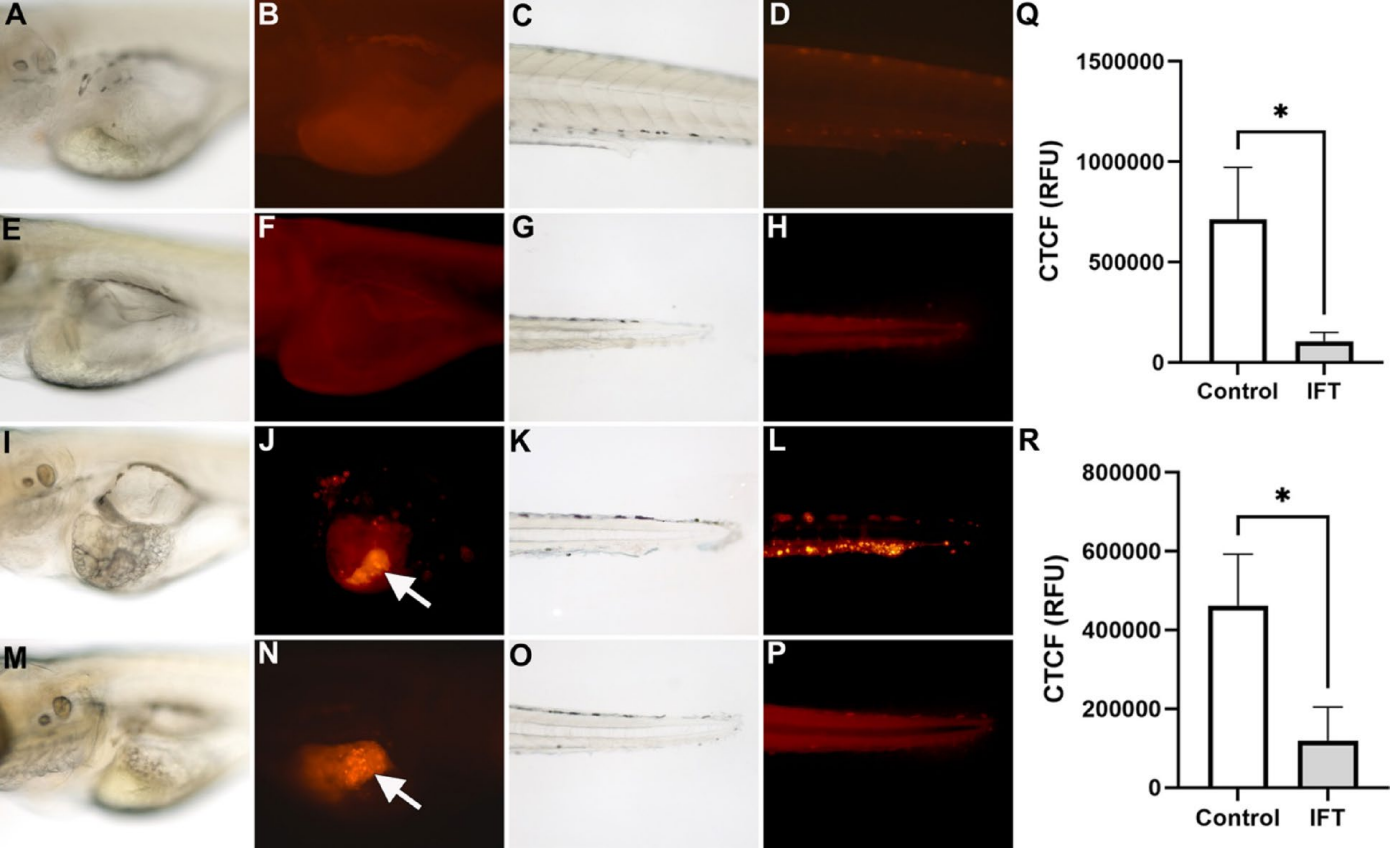
Ifetroban blocks dissemination of MDA-MB-231 xenografts in live zebrafish embryos.** A**-**H** Representative images of non-xenografted zebrafish larvae ± 5.25 M ifetroban treatment (A-D = Control, E-H = IFT). (**I**-**P**) Representative images of zebrafish larvae harboring MDA-MB-231 xenografts (injected in the yolk sac) ± 5.25 M ifetroban treatment (I-L = Control, M-P = IFT). **Q** Quantification of circulating tumor cell fluorescence after yolk sac injection of the xenografted cells. Data are represented as mean ± SEM (*n* = 17). The statistical significance is compared using two-tailed, unpaired t-tests. **R** Quantification of circulating tumor cell fluorescence after injection of the xenografted cells onto the circulation in the ductus venosus. Data are represented as mean ± SEM (*n* = 15). The statistical significance is indicated by asterisks and compared using two-tailed, unpaired t-tests. IFT-Ifetroban;* CTCF*  corrected total cell fluorescence, * RFU * relative fluorescence units. Arrow indicates clusters of red fluorescent MDA-MB-231 cells at the injection site (in the yolk sac)

To study the effects of TPr inhibition on TNBC migration in vivo, we took advantage of our TNBC (MDA-MB-231) xenograft zebrafish model [34]. TNBC cells (approximately 100 per embryo) were micro-injected into the yolk sac or into the ductus venosus (injection into the circulation) of 2 dpf zebrafish, exposed to ifetroban 3–5 dpf and analyzed at 5 dpf. No fluorescence is detected in either mock micro-injected embryos (Fig. 4A-D) or in un-injected embryos exposed to 5.25 µM ifetroban (Fig. 4E-H). Note that the ifetroban exposed embryos did not display any abnormal growth or morphological defects. Embryos injected with MDA-MB-321 cells at 2 dpf show the development of a primary tumor at the site of injection (Fig. 4J arrow) and metastatic cells in the tail (Fig. 4L) of the embryo. MDA-MB-321 cells xenografted at 2 dpf and exposed to ifetroban (3–5 dpf) are still able to form a primary tumor at the site of injection (Fig. 4M-N), but no metastatic cells are detected in the tail (Fig. 4P) of the embryo. Figure 4Q shows the quantification of the number of metastatic cells in the trunk or the tail of TNBC xenografted embryos exposed to ifetroban. The graph in Fig. 4Q was generated with 3 different xenograft experiments for a total of 17 embryos. Figure 4R shows the quantification of the number of metastatic cells in the trunk or the tail of TNBC xenografted embryos when the injection was performed directly into the circulation by xenografting TNBC in the ductus venosus while exposed to ifetroban. The graph in Fig. 4R was generated with 3 different xenograft experiments for a total of 15 embryos. Taken together, these results show that ifetroban exposure at the appropriate dose can reduce or block TNBC metastasis without affecting the number of platelets, re-enforcing its potential as an antimetastatic TNBC agent.

### Ifetroban reduces tumor growth and metastasis of TNBC in immune-competent mice

**Fig. 5:**
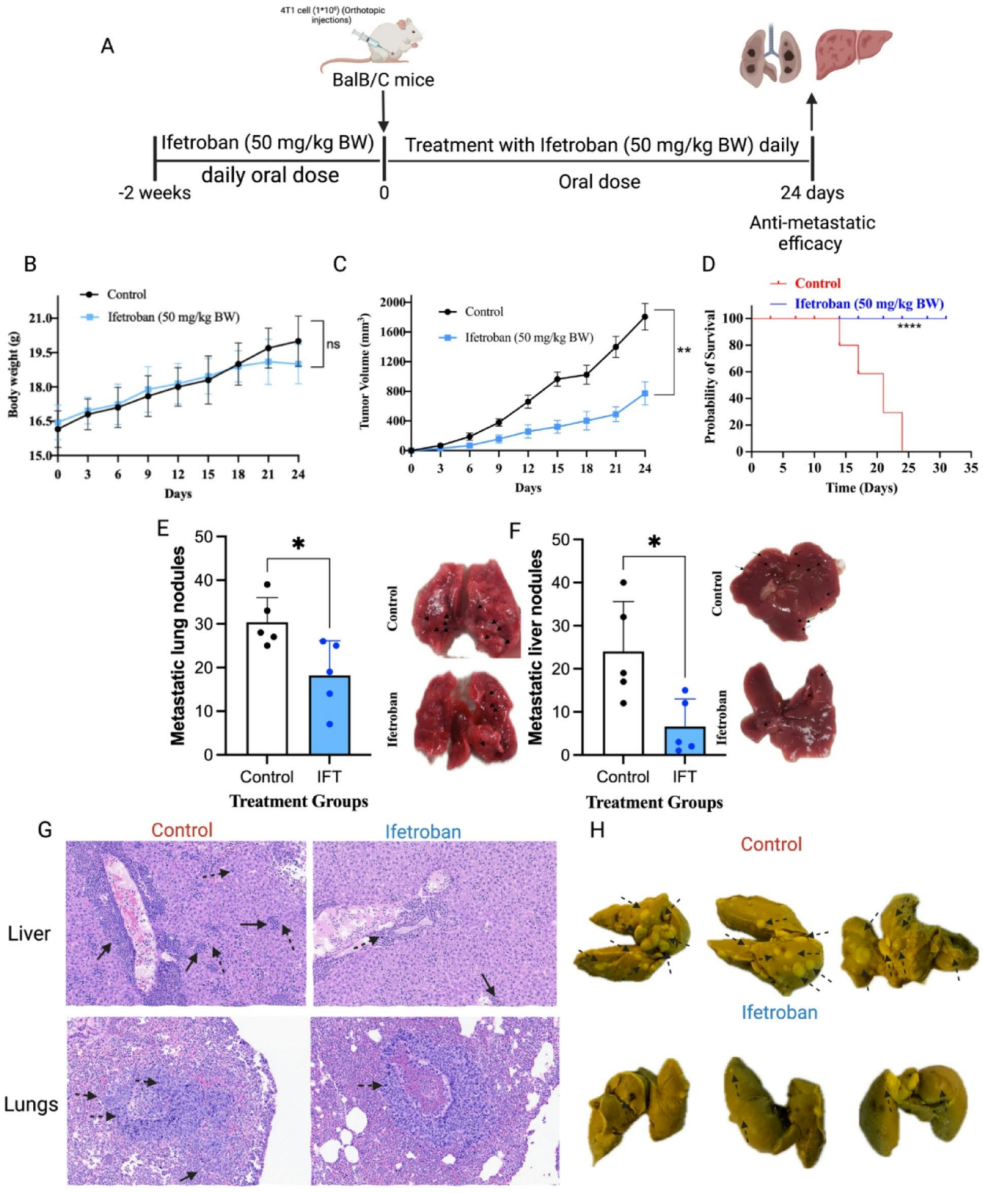
Ifetroban reduces growth and metastasis of 4T1 tumors in immune-competent mice.** A** Schematic representation of orthotopic 4T1 model and treatment protocol. **B** Representation of body weight changes with Ifetroban treatment over 38 days (*n* = 5). **C** Tumor volume changes with Ifetroban treatment over 38 days (*n* = 5). **D** Log-rank (mantel-cox) test was applied for survival analysis (*n* = 5). 4T1 metastatic nodules (solid black arrows) in **E** lungs and **F** liver of Balb/C mice treated with vehicle (*n* = 5) or ifetroban (*n* = 5). **G** Histology of lungs and liver (20X H&E) (*n* = 5). Perivascular inflammation with scattered perivascular (arrow) and sinusoidal (dotted arrow) metastatic cells. **H** Representative Bouin-stained lung tissues showing metastatic nodules. Data are represented as mean ± SD (*n* ≥ 5). The statistical significance is indicated by asterisks (*P < 0.05, **P < 0.01, ***P < 0.001 and ****P < 0.0001 vs. control group). IFT-Ifetroban

We further explored the impact of ifetroban on metastatic dissemination in murine models of TNBC. Orthotopic 4T1 mammary tumors were established in immune-competent Balb/C mice and monitored for tumor growth and metastasis to the lungs and liver while under treatment with ifetroban or vehicle control (Fig. 5A). Mice were treated daily with ifetroban for 2 weeks prior to tumor implantation, and treatment was continued during the 24-day experiment. This aggressive treatment regimen resulted in no significant change in body weight compared to vehicle-treated controls and minimal impact on blood components (Fig. 5B and Supplementary Table 1), indicating tolerability of the treatment schedule. Ifetroban treatment in this protocol resulted in a marked reduction in tumor volume of the ifetroban-treated animals compared to vehicle-treated controls (Fig. 5C). At the end of treatment on day 24, the tumors of ifetroban-treated mice were 61% the weight of tumors in untreated animals (Supplementary Table 2). Ifetroban also prolonged the overall survival of animals relative to those treated with vehicle control (****P < 0.0001, Fig. 5D).

Ifetroban given on the therapeutic schedule above significantly reduced metastatic nodules in the lungs (*P < 0.05, *n* = 5, mean reduction of 10 ± 2 nodules) and liver (*P < 0.05, *n* = 5, mean reduction of 20 ± 3 nodules) by 35% and 60%, respectively, compared to vehicle-treated controls (Fig. 5E-F). Histological analysis further revealed a reduction in the number of infiltrating metastatic neoplastic cells (dotted arrow) and attenuation of perivascular inflammation with scattered inflammatory foci (arrow) in the lung and liver of ifetroban-treated mice (Fig. 5G). Additionally, Bouin’s solution staining visually confirmed the decrease in the number of tumor colonies and lung weight in ifetroban-treated animals (Fig. 5H).

###  Ifetroban reduces metastasis in a human cell line-based model of TNBC

**Fig. 6:**
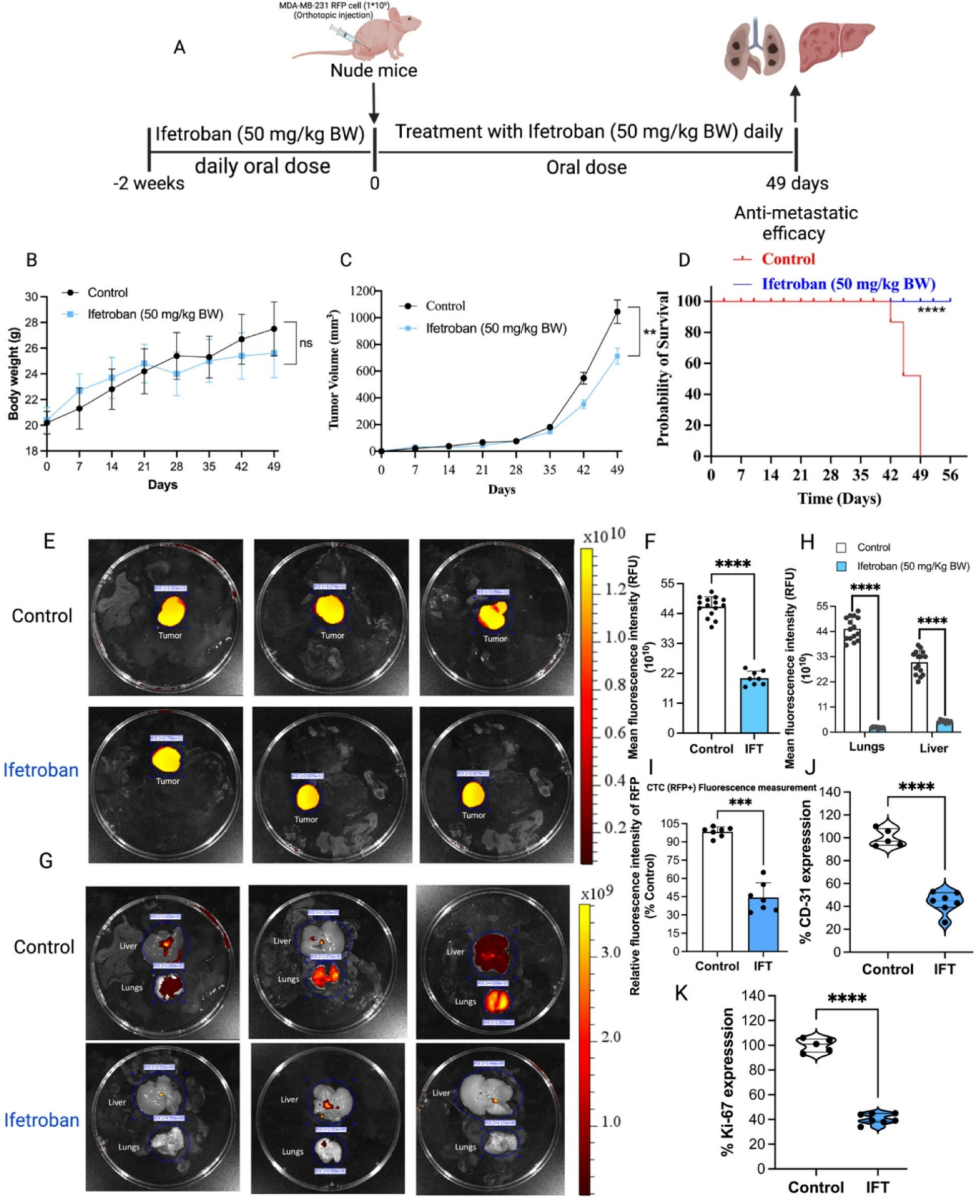
Ifetroban reduces circulating tumor cells, tumor vascularization, and proliferation, leading to reduced lung and liver metastasis of MDA-MB-231 tumors.** A** Schematic representation of orthotopic model and treatment protocol in athymic nude mice. **B** Representation of body weight changes with Ifetroban treatment over 49 days. **C** Tumor volume changes with Ifetroban treatment over 49 days (*n* = 10). **(D)** Log-rank (mantel-cox) test was applied for survival analysis. **E** Images of tumor RFP fluorescence. **F** Quantification of tumor fluorescence data in the vehicle (*n* = 15) or ifetroban (*n* = 8). **G** MDA-MB-231-RFP metastatic signal in lungs and livers of athymic nude mice treated with vehicle (*n* = 15) or ifetroban (*n* = 8). **H** Quantification of fluorescence data in the liver and lungs in the vehicle (*n* = 15) or ifetroban (*n* = 8). **I** MDA-MB-231-RFP fluorescence in the blood collected from vehicle- or ifetroban-treated animals (*n* = 7). **J** Image J-based quantification of CD-31 and **K** Ki-67 expression in tumor IHC sections (*n* = 5). Data are represented as mean ± SD (*n* ≥ 5). The statistical significance is indicated by asterisks (*P < 0.05, **P < 0.01, ***P < 0.001 and ****P < 0.0001 vs. control group). IFT-Ifetroban

To further validate the antimetastatic efficacy of ifetroban, we conducted additional studies in athymic nude mice harboring orthotopic xenografts of MDA-MB-231-RFP cells according to the same daily treatment course of ifetroban beginning two weeks prior to tumor cell inoculation (Fig. 6A). Again, we saw that this aggressive treatment regimen with ifetroban resulted in no significant change in mouse body weight (Fig. 6B), and we observed a modest decrease in the tumor volume of ifetroban-treated animals compared to vehicle control (Fig. 6C). In this model, ifetroban also significantly prolonged overall survival relative to control animals (****P < 0.0001; Fig. 6D). Tumor RFP intensity was quantified using IVIS imaging, where we observed a significant decrease in tumor burden in ifetroban-treated animals (****P < 0.0001, *n* = 10) as indicated by the significant reduction in RFP fluorescence from the tumors of these animals (Fig. 6E-F).

Using RFP fluorescence from the MDA-MB-231-RFP cells, we were able to monitor metastasis of the tumors to the liver and lungs by ex vivo IVIS imaging at the study endpoint. Lungs and livers harvested from ifetroban-treated mice showed 10x (****P < 0.0001, *n* = 8) and 6x (****P < 0.0001, *n* = 8) reduction in RFP fluorescence compared to vehicle controls, respectively (Fig. 6G-H), Indicating significant inhibition of metastasis to both sites. Histopathology of the lungs and liver confirmed a significant reduction in the metastatic foci in the ifetroban-treated group compared to the control (Supplementary Fig. 6A). We also monitored circulating tumor cells (CTCs) by measuring the RFP fluorescence in blood samples collected from mice at the study endpoint and found that ifetroban treatment reduced CTCs by 2.3x compared to vehicle-treated animals (****P < 0.0001, *n* = 7; Fig. 6I).

Ifetroban did not show any direct cytotoxic effects on MDA-MB-231-RFP and 4T1 cells (Supplementary Fig. 7A and 7B). Moreover, ifetroban promotes no cell death in zebrafish embryos, based on acridine orange staining of ifetroban-treated embryos compared to untreated embryos (Supplementary Fig. 8). Instead, IHC analysis of tumor sections displayed a reduced expression of CD-31 and Ki-67 (Fig. 6J and K**)** in ifetroban-treated tumor sections, indicating that ifetroban impacts tumor vasculature and proliferation to modulate tumor growth. Further, no change in the expression of cleaved caspase-3 (Supplementary Fig. 6B) was evidenced in ifetroban-treated mice tumors, depicting no effect on tumor cell apoptosis. These results align with prior studies that have described the role of TPr in the migration of endothelial cells and endothelial barrier function, and suggest that in addition to platelet-specific functions, ifetroban exerts some of its antimetastatic efficacy by modulating endothelial cells in the tumor microenvironment [30, 41].

*3.6. Ifetroban reduces metastasis in the absence of a primary tumor or when TBXA2R is deleted from tumor cells*.

**Fig. 7:**
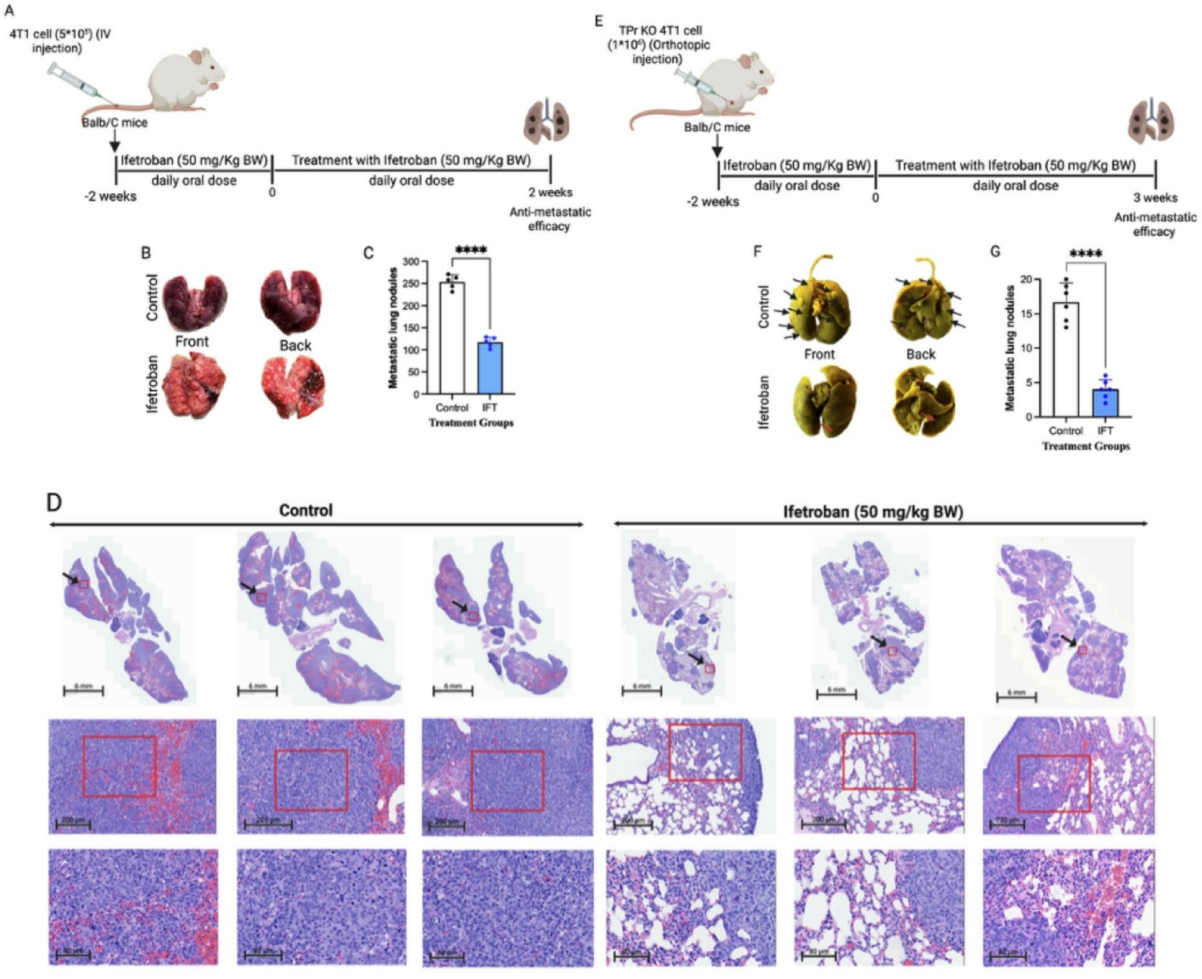
The impact of Ifetroban on 4T1 cell metastasis is independent of primary tumor growth or TPr expression in tumor cells.** A** Schematic representation of the experimental hematogenous metastasis model. **B** 4T1 metastatic nodules in Balb/C mice treated with vehicle (*n* = 10) or ifetroban (*n* = 14). **C** Quantification of metastatic lung nodules. **D** Representative lung tissues visualized by H&E staining (*n* = 5). **E** Schematic representation of the TPr KO 4T1 orthotopic model. **F** Bouin-stained TPr KO 4T1 metastatic lung nodules (solid black arrows) in Balb/C mice treated with vehicle (*n* = 6) or ifetroban (*n* = 9). **G** Quantification of metastatic lung nodules. Statistical significance is indicated by asterisks and compared using a two-tailed, unpaired t-test. IFT-Ifetroban

To further isolate the contributions of tumor cell TPr and platelet TPr to the antimetastatic efficacy of ifetroban, we conducted additional studies in an experimental hematogenous model of metastasis lacking a primary tumor and using *TBXA2R*-deleted tumor cells. BALB/c mice were injected intravenously with 4T1 tumor cells and treated with ifetroban according to the same daily treatment course beginning two weeks prior to tumor cell injection (Fig. 7A). Ifetroban given on the therapeutic schedule above reduced the number of metastatic nodules in the lungs by 67% compared to vehicle-treated controls (****P < 0.0001, *n* = 10, mean reduction of 175 ± 28 nodules; Fig. 7B-C**)**. Histological analysis further revealed a marked reduction in tumor burden in the lungs of ifetroban-treated mice compared to untreated mice (Fig. 7D **and Supplementary Table 3**). Next, we established orthotopic mammary tumors with *TBXA2R*-deleted 4T1 tumor cells (TPr KO 4T1 cells) and commenced treatment with ifetroban under the same protocol (Fig. 7E). Ifetroban treatment in this model reduced metastatic lung nodules by 72% (****P < 0.0001, *n* = 6, mean reduction of 16 ± 2 nodules) compared to vehicle-treated controls (Fig. 7F-G). Bouin’s solution staining visually confirmed the decrease in tumor colonies in ifetroban-treated animals (Fig. 7F). In sum, ifetroban retained its antimetastatic efficacy when tumor cells were injected directly in the bloodstream and when used to treat cell line-based tumors with TPr deleted from the tumor cell compartment.

## Discussion

In this study, we provided evidence that ifetroban reduces metastasis by inhibiting TPr signaling in platelets and interfering with the interaction of platelets and circulating tumor cells. Blocking TPr with ifetroban was sufficient to prevent platelet activation and platelet-tumor cell adhesion in cellular co-cultures. Ifetroban effectively reduced metastasis of TNBC cell lines in a zebrafish model of metastasis, and in murine models of TNBC. Moreover, treatment of mice with ifetroban potently inhibited metastasis in the absence of a primary tumor and when TPr was deleted from tumor cells, suggesting that platelet TPr inhibition was responsible for the antimetastatic efficacy observed of ifetroban. These findings directly underscore the role that TPr signaling can have in the metastatic cascade and provide justification for the further pursuit of ifetroban as an antimetastatic agent in the treatment of TNBCs.

Decades ago, an association between the depletion of platelets and reduced metastasis of cancers was demonstrated [5, 42–44]. Since these early observations, links between platelet activation, platelet degranulation, platelet-tumor cell binding, and metastasis have been well-documented [11, 42, 43, 45]. Thus, antiplatelet agents such as aspirin and others have shown promising antimetastatic potential in a range of tumor models [22, 29, 30, 45, 46]. Aspirin treatment markedly decreases the number of circulating tumor cells and platelet-tumor cell aggregates by blocking prostaglandin and TXA_2_ production in multiple tumor models [29, 45]. Moreover, there is substantial evidence supporting aspirin’s short-term effects on cancer mortality and its potential to reduce metastasis in various clinical cancer types [46–48], which is being tested further by the ongoing Add-Aspirin clinical trial (NCT02804815). Interestingly, while aspirin blocks both COX-1 and COX-2, recent studies suggest that the primary antimetastatic benefit of aspirin arises from its inhibition of COX-1 and the downstream production of TXA_2_ [45]. Additionally, Yang et al. recently showed that suppression of T cell immunity contributes to the antimetastatic effect of aspirin through reduced platelet-derived TXA_2_ [26]. This raises the premise that direct inhibition of TPr/TXA_2_ signaling with TPr-specific inhibitors such as ifetroban could retain the antimetastatic benefit of aspirin while avoiding off-target effects of COX-2 inhibition.

Our data indicate that the prevention of tumor cell-platelet interactions via treatment with the TPr inhibitor ifetroban is effective at reducing cancer metastasis. That said, the fact that ifetroban does not entirely inhibit platelet-tumor cell adhesion suggests that other processes could support the interaction as well. These processes, such as protease activated receptor (PAR) activation, P2Y12 receptor activation, and platelet integrin interactions could be targeted in combination with ifetroban in future studies [49]. Additionally, the activity of antiplatelet drugs in other cell types is shown to have potential antimetastatic effects as well. In two recent reports that *TBXA2R* is dysregulated in breast cancers [50, 51], malignancy was contributed primarily to increases in tumor cell proliferation, migration, or invasion. However, we have seen no such impact of ifetroban in this and previous studies, where ifetroban treatment of tumor cells showed no impact on tumor cell viability (Supplementary Fig. 7). It is likely though that ifetroban has some anti-tumor impact through other cells in the vasculature and tumor microenvironment. Paracrine TXA_2_ signaling induces vascular constriction and upregulates E-selectin and VCAM-1 via TPr on endothelial cells [28, 52]. Accordingly, activated endothelial cells recruit metastasis-promoting monocytes that establish a pre-metastatic niche [41, 53]. Moreover, we and others have shown that TPr disruption increases endothelial barrier function and prevents transendothelial migration of tumor cells in vitro [30]. Thus, while we show here that TPr can block the interaction of platelets and circulating tumor cells, it is likely that the antimetastatic efficacy of TPr inhibition is multifaceted and results from multiple mechanisms including the inhibition of platelet-tumor cell interactions and changes to endothelial barrier function.

Ifetroban likely impacts both the direct and indirect interaction of platelets with tumor cells. Direct adhesion of platelets to tumor cells influences multiple steps in the metastatic cascade, including survival in the circulation (i.e., prevention of anoikis), steric shielding of immune cell recognition, and improved adhesion of platelet-tumor cell aggregates to the vascular wall. Thus, blocking the direct interaction of platelets with tumor cells in circulation can leave circulating tumor cells more susceptible to clearance. We show here that ifetroban reduces the direct adhesion of platelets to tumor cells ex vivo in co-culture assays and reduces circulating tumor cell numbers in vivo. Therefore, it is likely that the disruption of direct platelet adhesion to tumor cells is at least partially responsible for the antimetastatic efficacy of ifetroban. However, the inhibition of platelet TPr with ifetroban could have indirect effects on tumor cell metastasis as well. Platelet degranulation downstream of TPr is known to produce soluble mediators that contribute to tumor cell EMT, tumor angiogenesis and endothelial barrier function, and immune modulation [11, 12, 17–19]. Based on recent work on the TPr/TXA_2_ axis in cancer metastasis, it appears likely that the antimetastatic efficacy of ifetroban is also due at least in part to changes in tumor angiogenesis/endothelial function and immune modulation. For example, reduced platelet activation could decrease the release of pro-angiogenic factors such as VEGF and TGF-β, thereby limiting tumor vascularization and growth [54]. Recent studies have highlighted the role of TXA_2_ in suppressing anti-tumor immunity [26, 29]. TXA_2_ can inhibit the activity of cytotoxic T cells and natural killer (NK) cells, which are essential for immune surveillance against tumor cells. By blocking TPr, ifetroban may enhance anti-tumor immunity by restoring the function of these immune cells. Our results confirm disruption of the direct interaction of platelets with tumor cells here. However, we cannot rule out the contribution of other indirect processes such as tumor angiogenesis and immune cell modulation as well.

The zebrafish model is now an established in vivo model to perform breast cancer cell xenografts [55], including TNBC cell xenografts [55, 56]. However, this study is the first to report a platelet/TNBC cell interaction in vivo in zebrafish larvae. As the zebrafish is becoming a more and more popular high throughput in vivo system for drug screening [57], we anticipate that xenograft models of various cancer cell types could be used in the near future to identify other compounds that can prevent platelet/cancer cell interactions in vivo and identify novel pathways that can play an active role in this phenomenon. Moreover, antiplatelet drug screening is very advanced in zebrafish research especially from a coagulation standpoint. The genetics of thrombogenesis is also well established in zebrafish models [58] and several groups have tested antiplatelet drugs in this model. These results suggest the future utility of zebrafish models in studying thrombogenesis and for the identification of novel drugs that affect platelet activity in the context of cancer and metastasis research as well. That said, cross-species dose comparisons between humans, mice, and zebrafish are limited by differences in drug administration routes and the lack of pharmacokinetic (PK) data, especially in zebrafish. In humans and mice, precise dosing via oral or injectable routes is supported by established PK measurements (e.g., blood concentration, AUC). In contrast, zebrafish, especially embryos and larvae, are typically exposed via immersion, where drug uptake is influenced by factors like skin permeability, water conditions, and developmental stage. This makes internal exposure levels unknown and variable. Moreover, the small size of zebrafish prevents their routine blood sampling, limiting PK profiling. Without data on absorption, distribution, metabolism, and excretion, it is difficult to determine whether observed effects are due to pharmacologically relevant exposure or nonspecific toxicity. These differences hinder reliable dose extrapolation and complicate the interpretation of efficacy or toxicity across species. As a result, findings in zebrafish may not directly translate to mammalian systems without additional PK or modeling data.

As blocking platelet-tumor cell interactions with antiplatelet agents such as ifetroban would represent a new clinical paradigm in the management of cancer metastasis, it is important to consider how best to deploy these agents. We chose the aggressive treatment regimen used here to determine that the TXA_2_/TPr pathway is critical for the initial adherence of platelets to CTCs and their survival within the vasculature events that can occur early upon tumor cell introduction. However, studies have also shown the efficacy of agents targeting this pathway in more clinically relevant protocols mimicking neoadjuvant and adjuvant therapy [29]. We, and others, have shown antimetastatic potential for antiplatelet agents in models of melanoma, colon cancer, lung cancer, pancreatic cancer, breast cancer, and others [29, 30, 34, 45]. Since it appears that platelets play a role in dissemination across many different tumor types, more work is needed to define which cancer types may respond best to antiplatelet agents. Our study provides a strong rationale for pursuing these agents in the context of TNBC. The appropriate window of treatment will be another important translational consideration for this new therapeutic approach. An ongoing clinical trial is evaluating the safety and efficacy of oral ifetroban given after completion of all planned therapy for 12 months to treat cancers (across multiple tumor types) with high risk of recurrence [59]. Future trials may also consider treatment in the neoadjuvant window for cancers such as TNBC where it is common practice to receive neoadjuvant therapy prior to surgery. The impact of these agents on the immune system is another important consideration to be studied further in future work. Here, we observed significant decreases in the number of total WBCs and monocytes in ifetroban-treated animals, while a trend toward reduction of neutrophils and lymphocytes was also observed. Since these were animals with advanced metastatic disease, one plausible explanation for the reduction in WBCs is the reduced tumor burden found in those animals. However, other reports have recently shown the impact of TXA_2_ on immune modulation. Therefore, it is possible that a lack of TXA_2_ in circulation caused a decrease in overall WBCs. This would need to be confirmed with additional experiments in future studies. Finally, it should be considered that our current data suggests ifetroban treatment likely will not eliminate existing primary tumors or metastases. Rather, ifetroban might prevent or decrease the spread of tumor cells to secondary and tertiary sites and should be given in combination with existing cytotoxic- or immuno-therapies.

## Conclusion

We performed studies in vitro and in multiple species to evaluate ifetroban as an antimetastatic agent in TNBC. In cell culture, platelets adhered readily to TNBC cell lines and this adhesion was increased further by activating TPr with U46619. In contrast, TPr inhibition with ifetroban reduced platelet-tumor cell adhesion in both the presence and absence of TPr stimulation by U46619. We further assessed the impact of ifetroban on platelets and disseminating tumor cells in zebrafish models which enabled high-resolution fluorescence imaging. Here, we observed reduced metastasis of MDA-MB-231 cells both from primary tumor sites established in the yolk sac and when injected directly into the circulation. Finally, we confirmed these results in two orthotopic murine models of TNBC. In these models, we demonstrated TPr inhibition, reduced platelet activation, reduced numbers of CTCs, and inhibition of tumor growth and metastasis to the lungs and liver. TPr inhibition in platelets appears to be a prominent mechanism of ifetroban’s antimetastatic activity, particularly since ifetroban prevents metastasis in the absence of a primary tumor or in *TBXA2R* deleted cells. However, modulation of TPr/TXA_2_ signaling in endothelial cells and immune cells likely contribute to the multifaceted mechanism of ifetroban in preventing tumor metastasis as well. Overall, our findings provide compelling evidence that ifetroban can be redirected from cardiovascular applications to be used as an antimetastatic agent in TNBC, where metastasis is a leading cause of mortality.

## Statement of significance

Ifetroban, a selective thromboxane A_2_-prostanoid receptor (TPr) antagonist, robustly inhibits triple negative breast cancer (TNBC) metastasis by modulating the TXA_2_ signaling pathway. Ifetroban decreases the formation of platelet-tumor cell aggregates, reduces the number of circulating tumor cells in the vascular compartment, and reduces metastasis to the lungs and liver. Therefore, ifetroban is a promising adjuvant therapy that could be applied to block metastatic dissemination of TNBCs.

## Supplementary Information

Below is the link to the electronic supplementary material.

Supplementary Material 1.

## Data Availability

All the raw data generated in this study are available upon request from the corresponding authors.
