# Supplementary material for: Targeting platelet-tumor cell interactions via thromboxane A2-prostanoid receptor blockade to reduce metastasis in triple negative breast cancer
Source: Exp Hematol Oncol. 2025 Nov 13;14:131. doi: 10.1186/s40164-025-00723-7 (PMC12613615; doi:10.1186/s40164-025-00723-7)
Supplement: Supplementary file 1 — Supplementary Material 1. [file 40164_2025_723_MOESM1_ESM.docx]

**Targeting platelet-tumor cell interactions via thromboxane A_2_-prostanoid receptor blockade to reduce metastasis in triple negative breast cancer**

*Veeresh Toragall^1^, Ann C. Rester^1^, Salma Begum^4^, Oluwaseyi T. Shofolawe-Bakare^2^, Kenneth Hulugalla^1,3^, Jerry D. Monroe^4,5^, John P. Bentley^6^, Yann Gibert^4,5*^, and Thomas A. Werfel^1, 2, 3, 5*^*

^1^Department of Biomedical Engineering, University of Mississippi, University, MS, USA

^2^Department of Chemical Engineering, University of Mississippi, University, MS, USA

^3^Department of BioMolecular Sciences, University of Mississippi, University, MS, USA

^4^Department of Cell and Molecular Biology, Cancer Center and Research Institute, University of Mississippi Medical Center, Jackson, MS, USA

^5^Cancer Center and Research Institute, University of Mississippi Medical Center, Jackson, MS, USA

^6^Department of Pharmacy Administration, University of Mississippi, University, MS, USA,

^$^Current address: Department of Medical and Molecular Genetics, Indiana University, Indianapolis, IN 46202

*Co-Corresponding Authors:

Thomas A. Werfel

Email: [tawerfel@olemiss.edu](mailto:tawerfel@olemiss.edu)

Address: A301 Brevard Hall, University, MS 38677

Yann Gibert

Email: [ygibert@umc.edu](mailto:ygibert@umc.edu)

Address: 2500 North State Street, Jackson, MS 39216

Conflict of Interest Statement: Cumberland Pharmaceuticals provided ifetroban for the studies as part of a research agreement between Cumberland and the University of Mississippi.

**
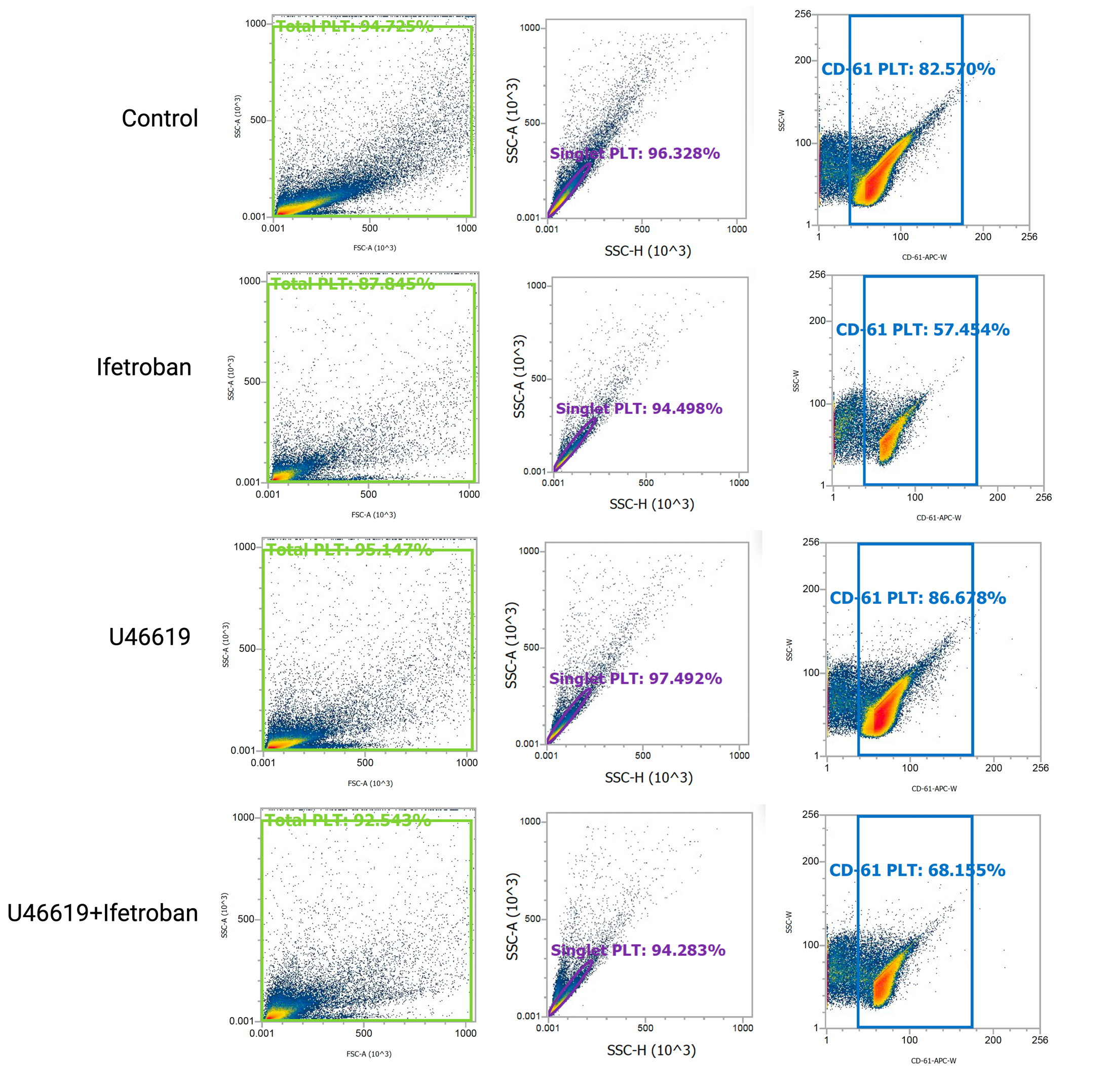
**

**Supplementary Figure 1.** Representative gating strategy for CD-61 expression quantification by flow cytometry.


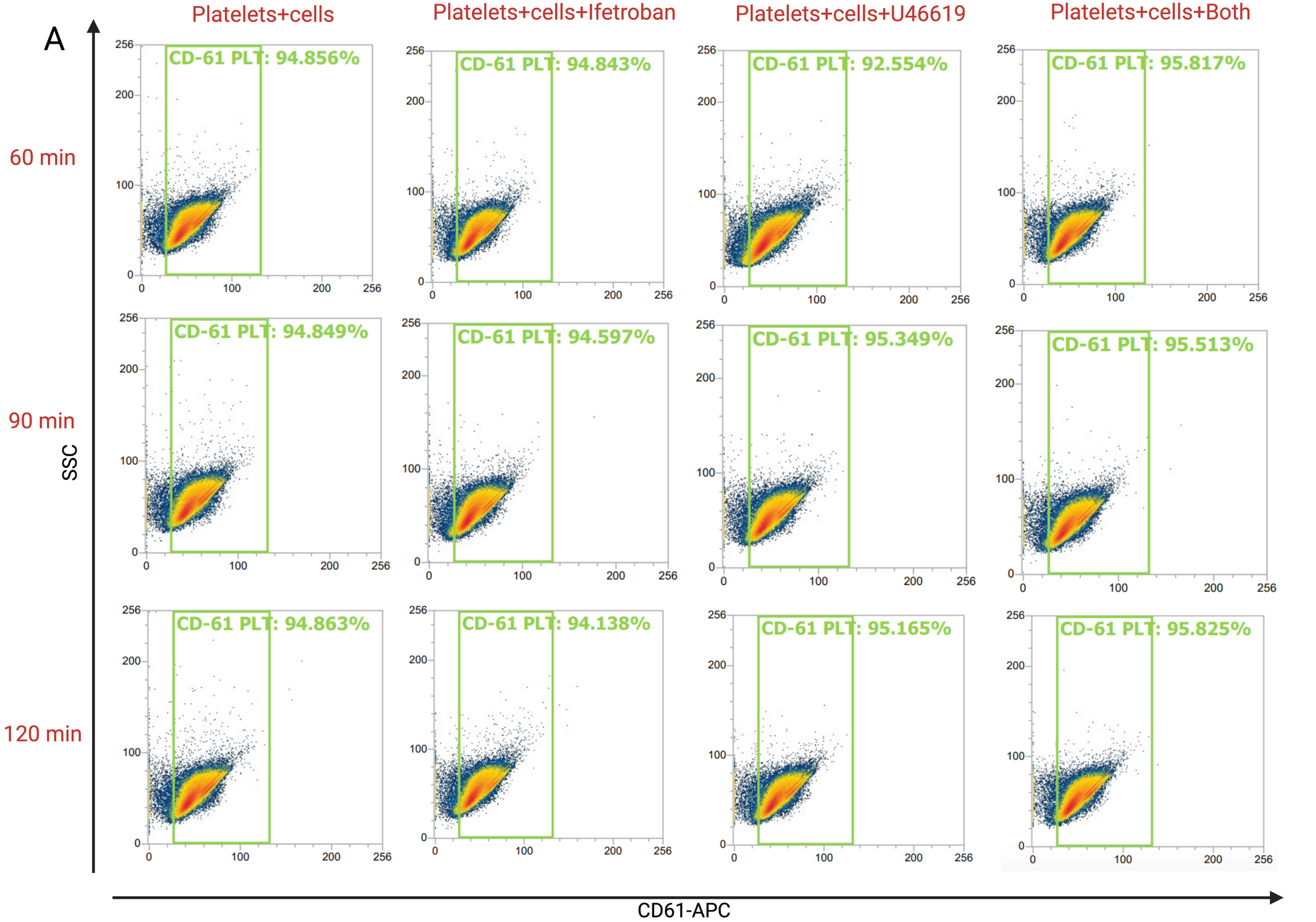


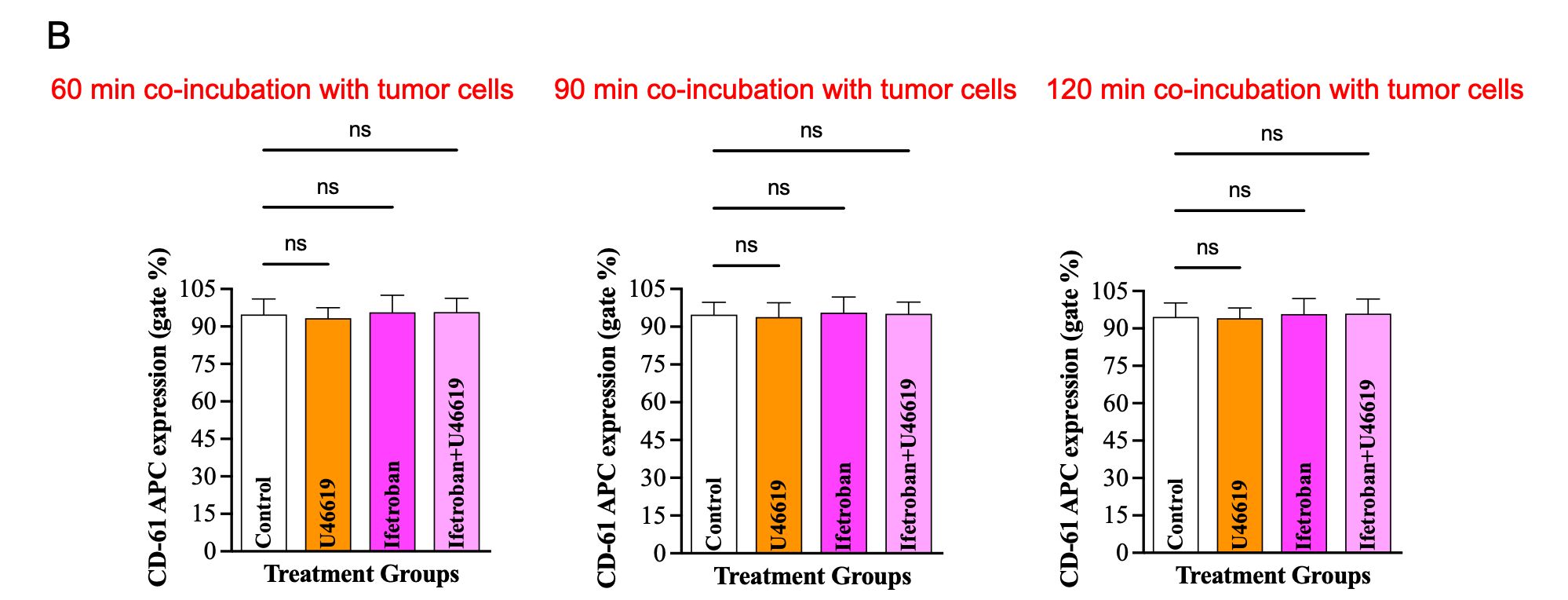


**Supplementary Figure 2.** **(A)** Representative two-parameter histograms of SSC vs. CD61-APC used to quantify CD-61 expression in platelets incubated with ifetroban pre-treated (M3) tumor cells. Tumor cells were pre-treated with ifetroban for 60 mins, followed by co-incubation of tumor cells and platelets for 60, 90, and 120 mins. **(B)** Quantification of %CD-61+ cells after 60 min, 90 min, and 120 min co-incubation of platelets and ifetroban pre-treated tumor cells. Data are represented as mean ± SD (n=5). The statistical significance was analyzed using one-way ANOVA. ns-non-significant

**
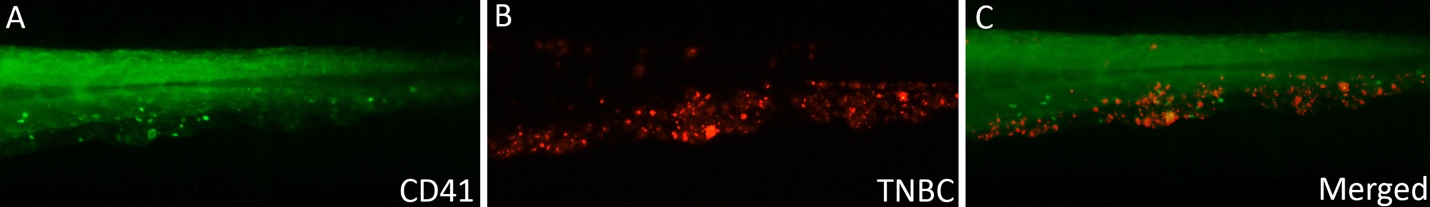
**

**Supplementary Figure 3.** Representative images of platelets (CD41) and MDA-MB-231 tumor cells (TNBC) directly interacting in zebrafish embryos.

**Supplementary Figure 4.** Treatment with 6.25 µM of ifetroban and below does not alter CD41 positive platelet numbers. **(A)** Brightfield image of control treatment sample at 120 hpf. **(B)** Fluorescence image of control treatment sample showing CD41 positive cells at 120 hpf. **(C)** Brightfield image of 5.25 µM ifetroban treated sample at 120 hpf. **(D)** Fluorescence image of 5.25 µM ifetroban treated sample at 120 hpf. **(E)** Brightfield image of 6.25 µM ifetroban treated sample at 120 hpf. **(F)** Fluorescence image of 6.25 µM ifetroban treated sample at 120 hpf. N=7.


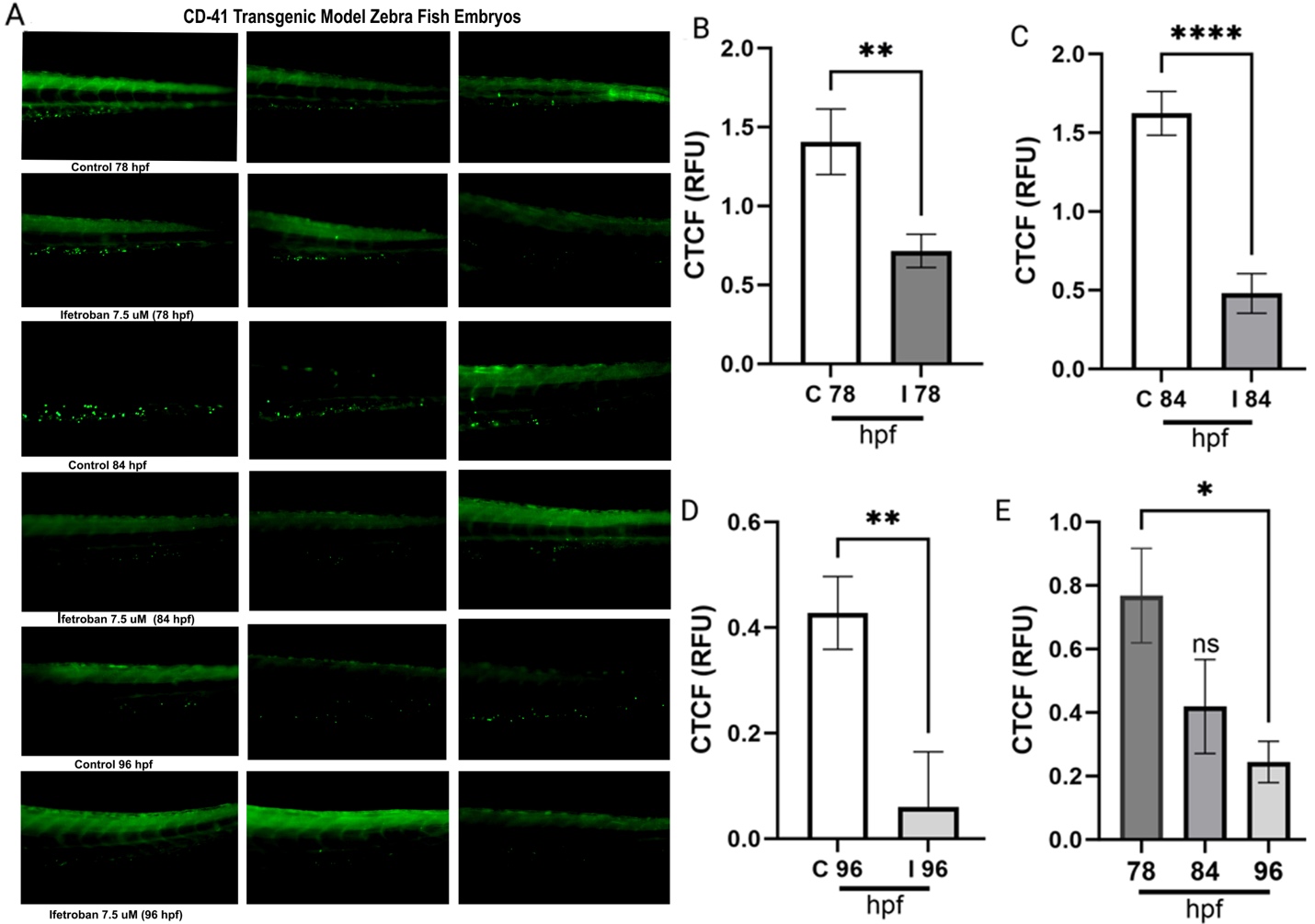


**Supplementary Figure 5.** CD41 expression in zebrafish embryos is depleted at 7.5 μM dose of ifetroban. (A) Representative images of CD41 transgenic zebrafish embryos ± 7.5 μM ifetroban treatment at 78, 84, and 96 hpf. (B-D) Quantification of CD41 fluorescence signal at 78, 84, and 96 hpf in embryos treated ± 7.5 μM ifetroban. (E) CD41 fluorescence signal over time in ifetroban-treated (7.5 μM) zebrafish embryos. Data are represented as mean ± SD (n=17). The statistical significance is indicated by asterisks (*P < 0.05, **P < 0.01, ***P < 0.001 and ****P < 0.0001 vs. control group) and compared using two-tailed, unpaired t-tests. C-Control, I-Ifetroban**.**

**Supplementary Table 1.** Effect of Ifetroban on Hematological parameters in mice after 25 days treatment. Data are represented as mean ± SD (n=5). The statistical significance is indicated by asterisks (*P < 0.05, **P < 0.01, ***P < 0.001 and ****P < 0.0001 vs. control group) compared using a two-tailed, unpaired t-test. IFT-Ifetroban

| **Parameters/Groups** | **Control** | **IFT** |
| --- | --- | --- |
| RBC (M/uL) | 6.9 ± 0.8 | 7.1 ± 0.9 |
| WBC (K/uL) | 710.6 ± 86.8 | 556.5 ± 67.8^**^ |
| HGB (g/dL) | 10.6 ± 0.4 | 10.5 ± 1.1 |
| MCV (fL) | 55.3 ± 1.2 | 54.0 ± 1.0 |
| MCH (pg) | 15.5 ± 1.3 | 14.9 ± 0.9 |
| MCHC (g/dL) | 28.0 ± 2.0 | 27.7 ± 1.3 |
| Platelet Count (K/uL) | 780.7 ± 120.7 | 763.0 ± 66.8 |
| MPV (fL) | 8.3 ± 0.4 | 8.4 ± 0.6 |
| Absolute Reticulocyte (K/uL) | 393.7 ± 69.5 | 298.3 ± 38.7 |
| Neutrophil (/uL) | 400387.7 ± 69780.7 | 31639.0 ± 83912.0 |
| Lymphocyte (/uL) | 290208.3 ± 16985.9 | 234396.0 ± 30763.5 |
| Monocyte (/uL) | 19970.7 ± 2043.4 | 5565.0 ± 678.1^***^ |
| Neutrophil (%) | 57.7 ± 12.2 | 56.3 ± 8.5 |
| Reticulocyte (%) | 5.8 ± 1.1 | 4.2 ± 0.2 |
| Lymphocytes (%) | 39.0 ± 13.5 | 42.7 ± 8.5 |
| HCT (%) | 38.1 ± 3.8 | 37.9 ± 4.7 |
| Monocytes (%) | 3.3 ± 0.35 | 1.0 ± 0.01^*^ |

**Supplementary Table 2.** Tumor weight and relative tumor weight after 25 days of treatment of Ifetroban in 4T1 tumor-bearing mice. Data are represented as mean ± SD (n=5). The statistical significance is indicated by asterisks (*P < 0.05 vs. control group) and compared using a two-tailed, unpaired t-test. IFT-Ifetroban

|  | **Tumor weight (g)** | **Relative Tumor weight (%)** |
| --- | --- | --- |
| **Control** | 2.73 ± 0.98 | 14.18 ± 1.44 |
| **IFT** | 1.65 ± 0.54^*^ | 8.85 ± 2.31^*^ |


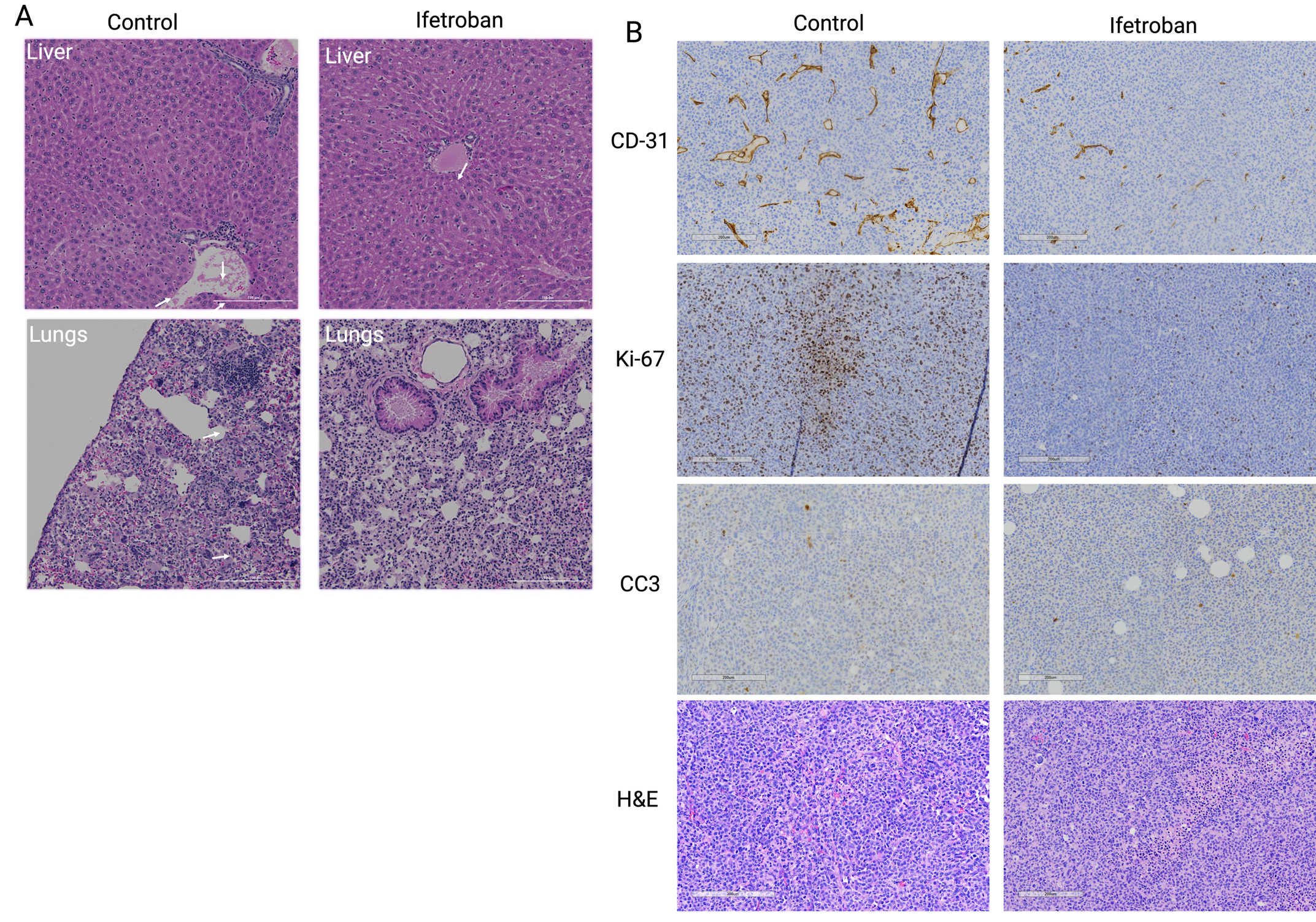


**Supplementary Figure 6.** (A) H&E staining of lungs and liver to examine the metastatic nodules. Arrows show neoplastic tumor cells. (B) IHC staining of excised tumors for CD31, Ki-67, CC3, and H&E after treatment with ifetroban and vehicle (4% sucrose) in athymic nude mice models.


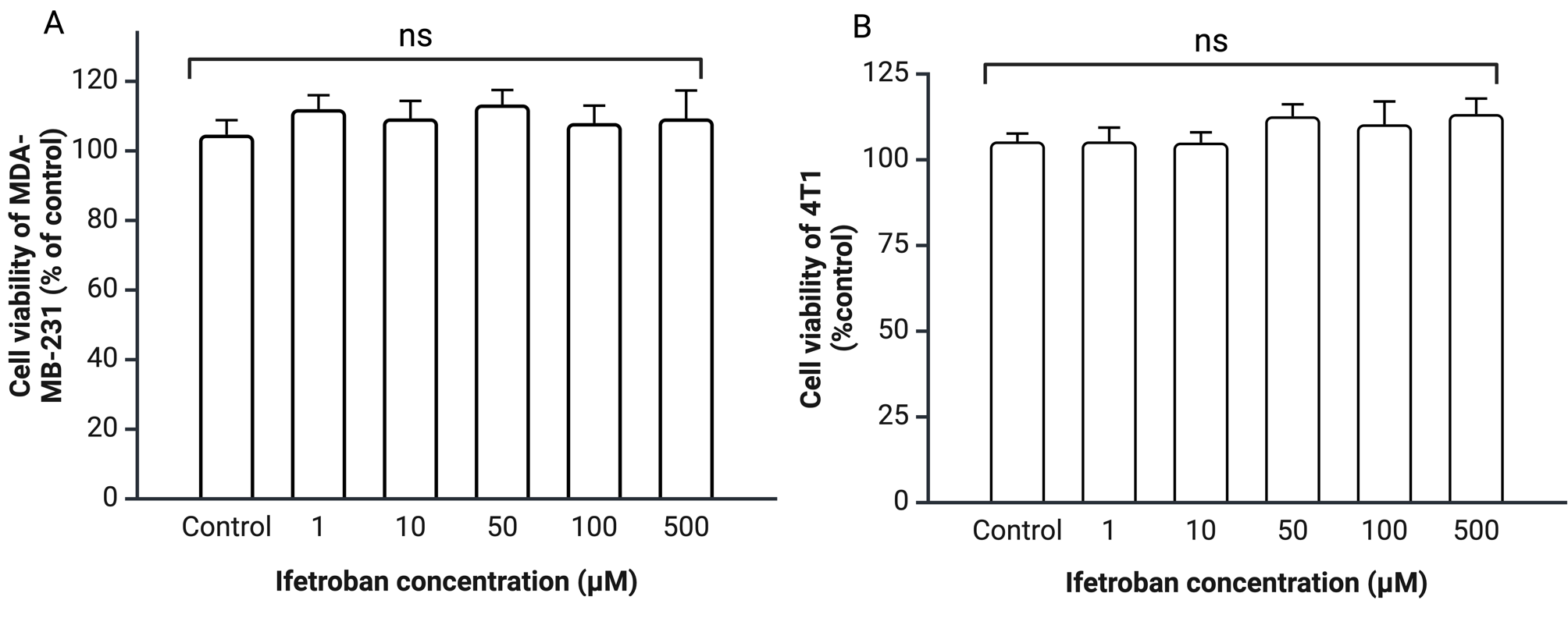


**Supplementary Figure 7. (A)** 24 h cell viability of MDA-MB-231-RFP cells treated with different concentrations of Ifetroban (n=3). **(B)** 24 h cell viability of 4T1 cells treated with different concentrations of Ifetroban (n=3). Data are represented as mean ± SD (n ≥ 3) and compared using one-way ANOVA. ns-non-significant


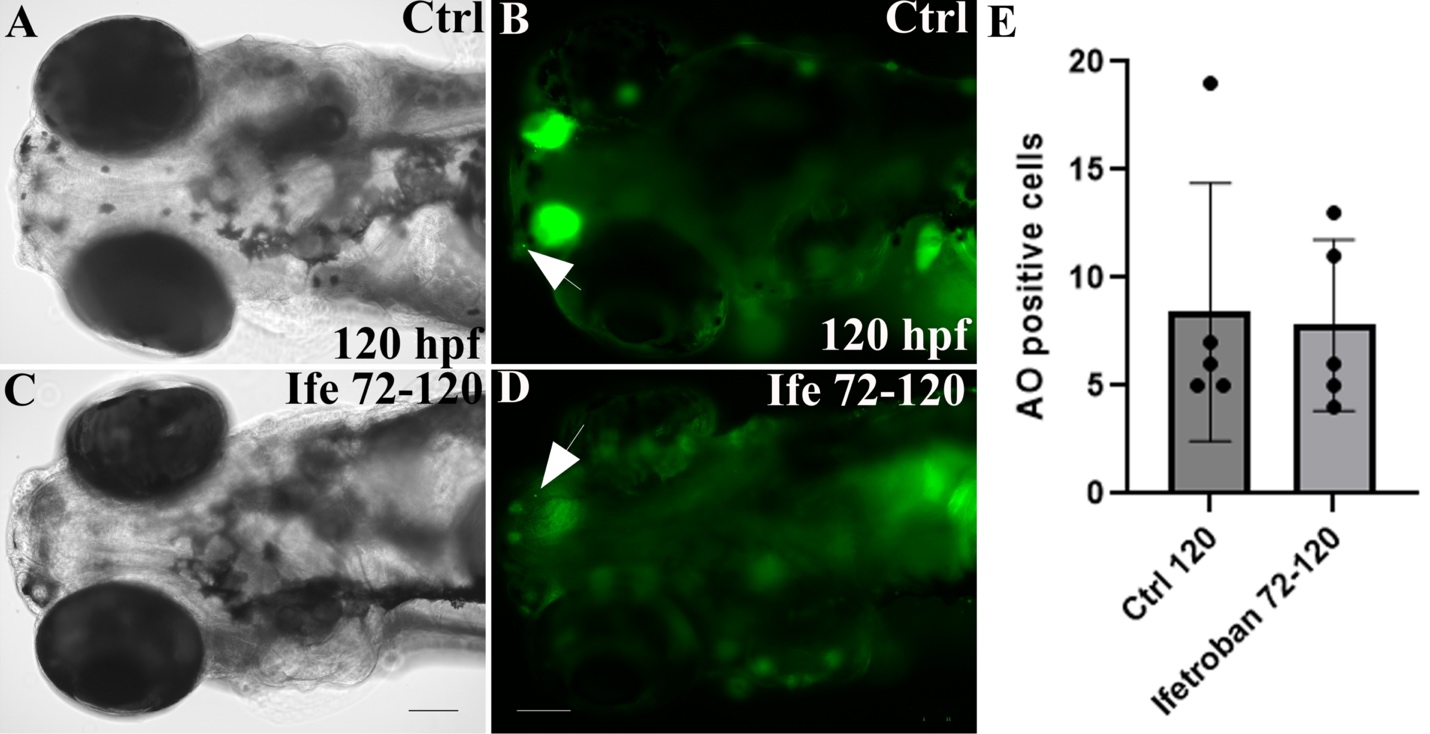


**Supplementary Figure 8.** Ifetroban does not promote cell death in zebrafish embryos. **(A)** Brightfield image of control treatment sample at 120 hpf. **(B)** Fluorescence image of control treatment sample showing acridine orange positive cells at 120 hpf. **(C)** Brightfield image of ifetroban treated sample at 120 hpf. **(D)** Fluorescence image of ifetroban treated sample at 120 hpf. **(E)** Comparison of acridine orange positive cells in control and ifetroban treated samples. Data are represented as mean ± SD (n=4). The statistical significance was analyzed using two-tailed, unpaired t-tests. AO = acridine orange; Ctrl = control; Ife = ifetroban; arrows indicate acridine orange positive cells.

**Supplementary Table 3.** Summary of Histopathological findings. Microscopic changes were graded as to severity utilizing a standard grading system, whereby: 0 = no significant change, 1= minimal, 2 = mild, 3 = moderate, and 4 = severe. International Harmonization of Nomenclature and Diagnostic (INHAND) Criteria standards are used as the basis for evaluation (<https://www.toxpath.org/inhand.asp>). The severity of the metastatic burden was graded utilizing the following grading system, whereby: 0 = no metastasis, 1= less than 25% of the tissue effaced by metastatic tumor, 2 = 25% to 50% of the tissue effaced by metastatic tumor, 3 = 50% to 75% of the tissue effaced by metastatic tumor, and 4 = 75% to 100% of the tissue effaced by metastatic tumor.

| **Control mice Lungs (n=5)** | | | |
| --- | --- | --- | --- |
| **Parameters** | **Abnormal** | **Mean Group Score** | **Mean Lesion Score** |
| Metastasis | 4.0 | 4.0 | 4.0 |
| Infiltrate mononuclear | 4.0 | 1.0 | 1.0 |
| Lysis/necrosis | 3.0 | 0.8 | 1.0 |
| Artifact | 0.0 | 0.0 |  |
| Sum-Scores | 11 | 5.8 | 6.0 |
| **Ifetroban treated mice Lungs (n=5)** | | | |
| **Parameters** | **Abnormal** | **Mean Group Score** | **Mean Lesion Score** |
| Metastasis | 4.0 | 2.5 | 2.5 |
| Infiltrate mononuclear | 4.0 | 1.0 | 1.0 |
| Lysis/necrosis | 0.0 | 0.0 | 0.0 |
| Artifact | 0.0 | 0.0 |  |
| Sum-Scores | 8.0 | 3.5 | 3.5 |
